# PhaseME: Automatic rapid assessment of phasing quality and phasing improvement

**DOI:** 10.1093/gigascience/giaa078

**Published:** 2020-07-24

**Authors:** Sina Majidian, Fritz J Sedlazeck

**Affiliations:** School of Electrical Engineering, Iran University of Science & Technology, Narmak, Tehran 1684613114, Iran; Human Genome Sequencing Center, Baylor College of Medicine, 1 Baylor Plaza, Houston, TX 77030, USA

**Keywords:** DNA sequencing, haplotype phasing, bioinformatics, quality assessment

## Abstract

**Background:**

The detection of which mutations are occurring on the same DNA molecule is essential to predict their consequences. This can be achieved by phasing the genomic variations. Nevertheless, state-of-the-art haplotype phasing is currently a black box in which the accuracy and quality of the reconstructed haplotypes are hard to assess.

**Findings:**

Here we present PhaseME, a versatile method to provide insights into and improvement of sample phasing results based on linkage data. We showcase the performance and the importance of PhaseME by comparing phasing information obtained from Pacific Biosciences including both continuous long reads and high-quality consensus reads, Oxford Nanopore Technologies, 10x Genomics, and Illumina sequencing technologies. We found that 10x Genomics and Oxford Nanopore phasing can be significantly improved while retaining a high N50 and completeness of phase blocks. PhaseME generates reports and summary plots to provide insights into phasing performance and correctness. We observed unique phasing issues for each of the sequencing technologies, highlighting the necessity of quality assessments. PhaseME is able to decrease the Hamming error rate significantly by 22.4% on average across all 5 technologies. Additionally, a significant improvement is obtained in the reduction of long switch errors. Especially for high-quality consensus reads, the improvement is 54.6% in return for only a 5% decrease in phase block N50 length.

**Conclusions:**

PhaseME is a universal method to assess the phasing quality and accuracy and improves the quality of phasing using linkage information. The package is freely available at https://github.com/smajidian/phaseme.

## Findings

Humans as well as many other organisms have a diploid genome, meaning that there are 2 homologous copies of every somatic chromosome inherited from mother and father. These copies include genomic variation including single-nucleotide variation (SNV) and structural variation (SV) [1–4]. Each variation represents a difference in a nucleotide(s) unique to each of the chromosome copies, also called haplotypes [5]. Thus, haplotypes represent the individual copy of each genomic element and need to be studied independently to investigate the effect of variations.

Haplotype phase information is essential to understand where a mutation occurs and to predict their interactions (i.e., if 2 SNVs are on the same DNA molecule) and their potential impact on genes and their expression and thus phenotypes. This is important for multiple applications and organisms. For humans, phasing plays an important role in, e.g., Mendelian diseases [6], cancer genomics [7–9], neurological diseases [10], genetic research, and other medical applications [11, 12]. As an example, compound heterozygosity shows the importance of phase information for relating genotype to phenotype. Numerous examples of disorders influenced by compound heterozygosity are known [11]. For example, thiopurine S-methyltransferase (*TPMT*) is a 27-kb gene located on chromosome 6p22.3. It is translated into an enzyme catalyzing S-methylation of thiopurine drugs [13]. These drugs are used as chemotherapeutic and immunosuppressant agents in lymphoid malignant neoplasms, leukemia, and inflammatory bowel disease. The enzyme activity is controlled by the genetic polymorphism of the gene. There are 2 SNPs (rs1800460 and rs1142345) where either are known to affect the activity of*TPMT* and cause missense changes [14]. Thus, it is important to know whether both SNPs exist, and if so whether they co-occur on the same haplotype (*cis*) leading to an inactivation of *TPMT* or not (*trans*). Patients with low *TPMT* activity are at higher risk of life-threatening severe myelosuppression and hematopoietic toxic effects when treated with conventional doses of mercaptopurine (or azathioprine) [15, 16].

Currently, we distinguish 4 different approaches to obtain phasing information [2]:

1. Wet-lab–based phasing is based on mainly 2 different methods: encapsulation and 3D structure capture [17]. One method is to extract chromosomes when cells are in metaphase and then microdissect them into subsets [18].
2. Population-based phasing uses linkage information derived from hundreds to thousands of individuals [19]. However, this approach misses rare and *de novo* variants [20]. Furthermore, it requires available population-based sequencing, which might not be available for many of the non-model organisms.
3. Parental methods [21] have the advantage to phase entire genomes but lack the ability to phase *de novo* mutations (i.e., important in many Mendelian diseases). Furthermore, they require sequencing of the parents, which can be more expensive and often not applicable.
4. Single individual haplotyping (SIH), or haplotype assembly, is the most comprehensive approach because it includes *de novo* mutations and rare variations, but it is often hindered by fragmentation [1]. HapCut2 [22] and WhatsHap [23] are the 2 most commonly used algorithms for this approach capable of utilizing Illumina, Pacific Biosciences (PacBio), Oxford Nanopore Technologies (ONT), 10X Genomics, and HiC information. In short, these approaches rely on aligned reads to a reference genome from which the SNVs and their genotypes are inferred. Subsequently, the SIH methods cluster the reads along the heterozygous SNV into 2 groups corresponding to the 2 haplotypes. The resulting VCF file reports SNVs with their phasing information, which includes the assignment of each SNV to a phase block. It is important to note that the relationship between individual phase blocks remains undetermined [2].

Based on the above information, SIH methods are important and necessary for a better understanding of the genome at hand. However, it remains tedious to impossible to assess the accuracy and even the performance of individual samples. Generally, all of them may be hindered by inaccurate results, which includes errors in the grouping of reads leading to incorrectly assigned SNVs (flip errors) or inaccurately joined haplotypes (switch errors). Currently phasing is often evaluated solely on the basis of its phasing length, e.g., N50. Phasing N50 is the minimum phase block length, where the sum of its phase blocks with all larger phase blocks spans ≥50% of the total phase length. Similar to assembly methods however, N50 does not represent the accuracy or quality of a result. In addition, most phasing methods do not provide a quality score to assess the reliability of the phasing itself, making it near impossible for users to assess the quality or correctness of their results.

To solve these problems and limitations we developed PhaseME, a method to automatically estimate the quality of the SIH results and report multiple statistics to enable a deeper understanding for a broad range of users. This is done on the basis of population data to assess the accuracy of common variations across the individual phasing information. Furthermore, PhaseME can detect phasing errors and highlight their locations. If desired, PhaseME continues to correct the detected phasing errors and generates a report of the improvement and impact of these changes. Thus, PhaseME is unique in its usability and application because it provides more insights into the phasing accuracy per sample. To the best of our knowledge, there exists only 1 other highly specialized tool, which requires population information together with Hi-C data to correct SIH phasing and does not provide insights in the phasing results [24]. In the following, we describe the features of PhaseME and its applications for different technologies based on SIH phasing for HG002. We assess the performance of PhaseME based on parental phasing information from Genome in a Bottle (GIAB) [25] across 5 different sequencing technologies. It is worth noting that parental data also have some limitations (e.g., *de novo* mutations); however, HG002 is a healthy individual that is well studied.

### PhaseME

**Figure 1:**
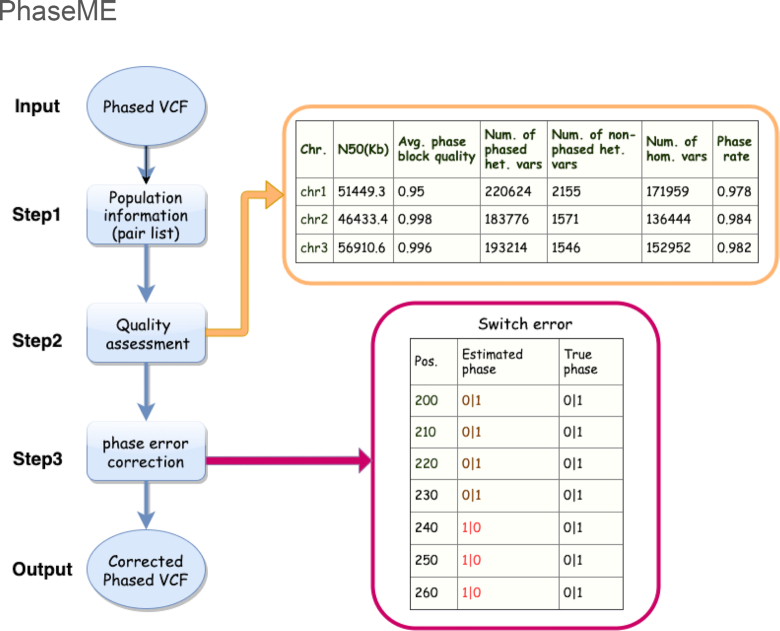
Summary of PhaseME. PhaseME consists of 3 steps: extracting population information, quality assessment, and phase error correction. Avg.: average; chr.: chromosome; het.: heterozygous; hom.; homozygous; num.: number; pos.: position; var: variant.

PhaseME reduces phasing errors by exploiting population information. Figure 1 gives an overview of the 3 main steps of PhaseME. First, PhaseME requires the phased SNVs VCF file for an individual obtained from an SIH method, which is compared to precomputed linkage information that is available per ethnicity (see the Methods section for details).

Second, PhaseME returns an in-depth quality assessment report of the phasing result to provide a detailed overview. PhaseME calculates the quality ratio across phase blocks based on the previously obtained linkage information. Here, for each phase block, we compute the ratio of SNVs with no conflict over all SNVs and report the mean per chromosome. Thus, 0 represents the lowest accuracy, while 1 indicates that everything is supported by the linkage information and more likely correct. PhaseME further reports the N50 of phase block length in kilobase pairs, the number of phased and non-phased heterozygous and homozygous variants, mean phase block quality, and phase rate (see the Quality assessment based on PhaseME subsection and Methods section for details).

Third, PhaseME corrects the previously identified SNVs that are in conflict with the linkage information. We distinguish small (2–20 bp) and large (≥21 bp) switch errors that represent a stretch of incorrectly phased SNVs, and thus PhaseME splits the existing phase block into 2 at the first conflicting SNV (see the section on error correction for details).

### Quality assessment based on PhaseME

As highlighted above, it is essential to obtain insights into the phasing quality. PhaseME is designed with this as its main application and to provide an easy-to-understand and comprehensive quality report across the phasing results. We outline the provided summaries below based on SIH results for HG002 from GIAB [26] (see Methods section).

We used PhaseME to compare and assess the quality of the phasing across ONT, PacBio continuous long reads (CLR), PacBio HiFi [27], 10x Genomics, and Illumina based on available linkage information (see Data description in Methods section). For each SNV, we consider the linkage information obtained from the 1000 Genomes Project of the population data if it is in conflict (i.e., mismatched) or in agreement (i.e., matched). Thus the higher the number of matched SNVs, the better is the phase quality of the phase block.

To inspect the haplotype length, PhaseME reports (i) N50 of phase block length in kilobase pairs, which highlights the overall length of the phasing; (ii) number of phased and non-phased heterozygous variants, which illustrates the completeness of the phasing; (iii) number of homozygous variants; (iv) the phase rate to indicate the proportion of phased regions for each chromosome; and (v) the mean phase block quality to indicate the agreement with the linkage information. The detailed definition of each criterion is provided in the Methods section. On the basis of this report, one can determine the phasing quality for each chromosome and phase block of the sample at hand. Each of these statistics is automatically generated and provided by PhaseME in the quality assessment report file.

**Figure 2:**
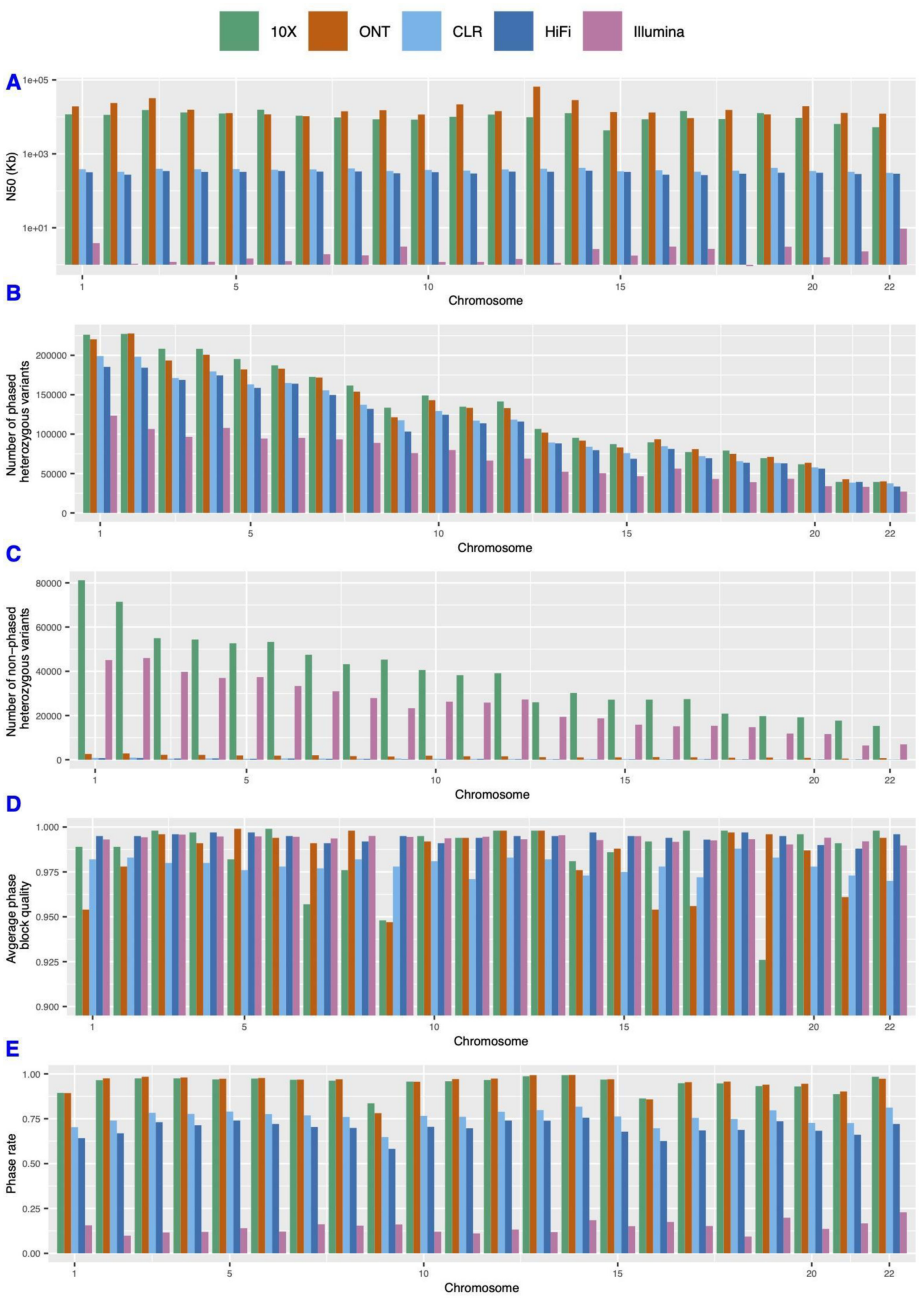
Phasing quality overview. Quality of reconstructed haplotypes for HG002 by WhatsHap across the different technologies: 10x Genomics, ONT, PacBio CLR, PacBio HiFi, and Illumina. (A) N50 of phase block length in kilobase pairs (in logarithmic scale). (B) Number of phased heterozygous variants for each technology, highlighting a higher number of heterozygous SNVs in general for 10x Genomics and ONT. (C) Comparison of the non-phased heterozygous variations along the chromosomes. (D) Mean phase block quality and (E) phase rate based on the 5 technologies.

As expected we observed the smallest N50 phase block length for Illumina (1.3 kb) and a high N50 for 10x Genomics (10.9 Mb), but interestingly even higher for ONT (15.6 Mb) (Fig. 2A). PacBio CLR (369.3 kb) or PacBio HiFi (314.3 kb) showed similar phase block N50. One likely reason is the longer read lengths of the technologies compared with PacBio. Interestingly PhaseME highlights a higher number of heterozygous SNVs for 10x Genomics (2,890,988) followed by ONT (2,807,291), whereas we observed a lower number of heterozygous SNVs for PacBio CLR (2,520,418), HiFi (2,418,009), and Illumina (1,522,191) (see Fig. 2B). For 10x Genomics the rate of non-phased heterozygous SNVs is also high in contrast to ONT (Fig. 2C). The results of PhaseME show that the mean phase block quality was the highest for HiFi (0.994), similar to Illumina (0.9936), followed by 10x Genomics (0.985), ONT (0.983), and CLR (0.978). We observed a lower phase quality for chromosome 9 when using ONT and 10x Genomics data (see Fig. 2D). However, we did not observe that this affects the same regions. We investigated the source of this error for ONT and 10x Genomics on chromosome 9. When we compared the SNV calling to the GIAB gold standard [28], we found lower precisions for the ONT (0.67) and 10x Genomics (0.71) call sets. This highlights a larger number of falsely called SNVs for ONT (64,496 SNVs) and 10x Genomics (61,392 SNVs), which may lead to confusions during the phasing, whereas CLR (56,799 SNVs), Illumina (44,932 SNVs), and HiFi (29,995 SNVs) had lower rates of false-positive SNVs (see Supplementary Table 3).

It is interesting to note that the PacBio CLR data show a lower N50 and a lower phase accuracy compared with ONT. However, this might be explained by the fact that CLR data are from 2015 and were generated with the RSII instrument for GIAB [25]. Another important observation is that the ONT data had the largest N50 phase length but were also quite accurate, being only 0.11 behind the HiFi reads, which were the most accurate but had an almost 60 times reduced N50. Another important metric for assessment of phasing completeness is phase rate, which represents the fraction of regions phased along the genome. Fig. 2E shows the results based on the PhaseME report across the technologies. 10x Genomics (0.947) and ONT (0.949) had the highest phase rate. This is followed by CLR (0.75), HiFi (0.69), and Illumina (0.145), probably strongly related to the molecule/read size and their higher number of heterozygous SNVs. Illumina (0.26) showed the highest ratio of non-phased heterozygous SNVs, followed by 10x Genomics (0.22) and ONT (0.01). For PacBio we observed a lower ratio with CLR (0.0034) and HiFi (0.0032) (see Fig. 2C).

### Phasing error correction

Phasing errors lead to the misassignment of mutations to the wrong haplotype. PhaseME aims to correct larger switch errors because the linkage data does not provide sufficient resolution to correct single flip errors. To detect switch errors, PhaseME considers mismatches indicated by upstream SNVs to improve the signal. PhaseME requires a minimum of 2 mismatches and considers the ratio between matching and mismatching linkage information for the SNVs. If this ratio (matches/mismatches) < 1 (by default), then PhaseME breaks the section in 2 separate phase blocks with the first conflicting SNV as the start of the new phase block.

We benchmark the error correction ability of PhaseME across different sequencing technologies (ONT, PacBio HiFi, PacBio CLR, 10x Genomics, and Illumina) on the basis of a male Ashkenazi proband, HG002 (NA24385) from the GIAB repository and compared the results to the parental phasing information based on Illumina SNVs [25] (see Benchmarking of phasing data **in**Methods section). Note that HG002 is not included in the population dataset (1000 G data) that was used to obtain the linkage information.

We also consider the Hamming error rate [29] to evaluate the phasing, which shows the fraction incorrectly phased over the total number of phased heterozygous variants (see Methods section). ONT (0.203) has the highest Hamming error rate followed by 10x Genomics (0.073), PacBio CLR (0.0389), PacBio HiFi (0.0098), and Illumina (0.00645) with the smallest Hamming error rate. This seems a bit contradictory to our previous results where ONT had a high average phasing quality (Fig. 2D). This is likely due to higher switch error rates in ONT (780) vs the other technologies PacBio CLR (497), PacBio HiFi (230), and 10x Genomics (50). Clearly, these errors are also related to the overall size of phase blocks where ONT (15.6 Mb) and 10x Genomics (10.9 Mb) had the longest N50. For short 2–20 bp switches, we observed the highest number of errors for Illumina (1,700) followed by CLR (1,452), 10x Genomics (1,356), ONT (698), and PacBio HiFi (455). We did not observe a correlation between the number of long switch errors and the length of phase blocks (Spearman correlation test *P*-value = 0.14 [ONT], 0.19 [10x Genomics], 0.69 [PacBio CLR], and 0.66 [PacBio HiFi]), which thus explains the different results (see Supplementary Fig. 1).

**Figure 3:**
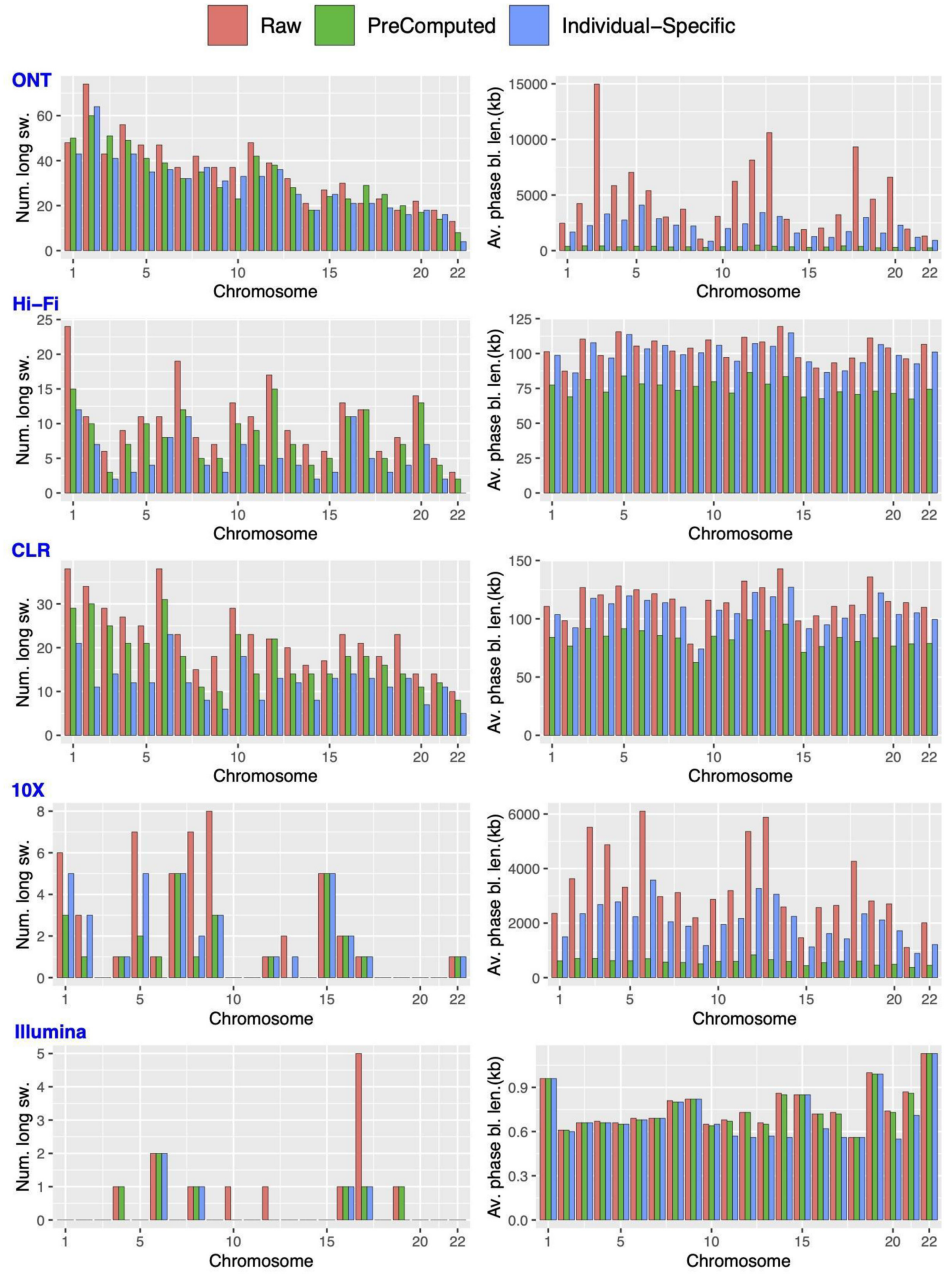
Phasing improvement. Comparison of raw haplotypes and improved haplotypes in terms of number of long switches (sw.) and average (av.) phase block length (bl. len.) (kb) for 5 datasets: ONT, PacBio HiFi, PacBio CLR, 10x Genomics, and Illumina. Precomputed with the allele frequency in the European populations (VCF tag: EUR_AF) greater than 0.01.

PhaseME overall reduced the phasing errors based on the linkage information. Fig. 3 shows the results of improving the phasing and effect on phase block length for PhaseME. For the Hamming error rate, we observed on average a 22.4% reduction in errors across the technologies based on the evaluation compared to the parental phasing information (see Methods). For ONT (34.3%), the reduction was the highest, followed by 10x Genomics (25.1%), PacBio HiFi (24.5%), PacBio CLR (20.8%), and Illumina (16.8%). Fig. 3 shows the improvement of long switch errors (≥21 SNVs). Here PhaseME reduced the error for the ONT data set from 780 down to 647 (17.1%). This resulted in a reduced N50 from 15.6 Mb down to 6.3 Mb. For CLR, PhaseME decreased the long switch errors by 46.0%, which led to a decrease of phasing N50 from 369.3 to 339.1 kb. For 10x Genomics we could improve the phasing by 23.5% with a reduced N50 from 10.9 to 5.9 Mb. The number of long switch errors for HiFi was reduced by 54.6% (see Fig. 3), which leads to a reduced N50 from 314.3 to 300.3 kb. For Illumina we observed a 61.5% decrease of long switch errors in return of 9.8% decrease in N50 (from 1.3 to 1.2 kb). Next, we evaluated PhaseME for short switches (2–20 bp). Here, the linkage data does not provide the resolution to improve most of them. Thus, the number of short switches is decreased for Illumina (47.0%), CLR (22.4%), HiFi (18.6%), and 10x Genomics (1.5%). However, our comparisons to the parents indicated that for ONT we actually introduced 6.6% of short switch errors (see Table 1). Thus, we provide parameters to adjust PhaseME (see Supplementary Table 2).

**Table 1:** Summary of results using PhaseME including short (2–20 bp) and long (≥21 bp) switches and the corresponding run time in minutes

Technology	Short switch		Long switch		Run time (min)	
	Raw	Change (%)	Raw	Change (%)	Quality assessment step	Error correction step
ONT	698	+6.6	780	−17.9	120	6
PacBio CLR	1,452	−22.4	497	−46.0	10	4
PacBio HiFi	455	−18.6	230	−54.6	9	4
10x Genomics	1,356	−1.5	50	−23.5	135	7
Illumina	1,700	−47.0	13	−61.5	467	18

A negative value indicates improvement, while a positive value represents an introduction of false cuts compared to the parental information. For short switches, we observed a lower performance due to the low resolution of linkage data compared to the long switch errors. For the runtime the quality assessment part takes longer as it includes the detection of the errors.

We implemented 2 modes to further ease the use of PhaseME for non-expert users. We recommend following the instructions to obtain linkage information for each sample. Nevertheless, to simplify the use of PhaseME, we also investigated the use of precomputed linkage maps. We computed the linkage map based on SNPs for a given ethnicity. Here we use the SNPs with the allele frequency in the European populations (VCF tag: EUR_AF) greater than 0.01. The number of long switch errors for PacBio HiFi and CLR are only improved by 22.6% and 20.9%, while the mean phase block length is 75.2 and 83.2 kb, respectively. For ONT PhaseME improved 10.6% of the number of long switch errors, leading to a mean phase block length of 355.1 kb. Further adjustments of the minimum allele frequency did not provide significant improvements (Supplementary Tables 1 and 2). Therefore, we recommend that only non-expert users use the precomputed linkage maps, but we suggest following our guidelines to obtain a sample-specific linkage map once larger errors are initially detected. PhaseME can compute a rapid quality assessment where the run time depends on the size and number of phase blocks. For these data sets, PhaseME took between 9 (HiFi) and 467 minutes (Illumina) to compute the quality assessment results (see Table 1). For the error correction steps, PhaseME is optimized to run multiple times with different parameters and only requires between 4 (HiFi or CLR) and 18 minutes (Illumina). The program was run on a Linux machine with 16 GB memory using a single CPU (AMD, 1.4 GHz) but typically requires ≤3 GB of memory. However, we note that using Shapeit to obtain linkage-based phasing information might have a higher memory consumption.

Additionally, PhaseME can also utilize the parental information to correct the sample phasing. Here PhaseME uses the SNV information of the parents to assign all overlapping SNVs of the offspring to the haplotype and vice versa for the maternal overlapping SNVs. We do not consider SNVs without overlap or overlapping both parental SNV sets. In contrast to the linkage mode, we only flip the SNVs according to the parental signal (see Supplementary Fig. 2). We did not benchmark the parental phasing because we are using this strategy as evaluation of the population-based phasing.

PhaseME represents a versatile and easy-to-use method to obtain insights into the phasing performance independent of the underlying sequencing technology. It allows non-expert users to gain valuable insights into the data set and the correctness of the phasing given an available linkage map. Here we have shown the performance of PhaseME based on HG002 across 5 different sequencing technologies, demonstrating a significant improvement over long switch errors. Smaller errors in phasing remain a challenge due to the lower resolution of linkage data. These can, however, be improved using parental phasing. Consequently, we did not attempt to correct single SNV phasing errors. To enable utility to a broader range of users we have provided precomputed linkage maps that can be used to obtain an initial improvement and insight, but we highly recommend that users compute the linkage map specific to their study/sample. PhaseME is capable of being run on multiple organisms and functions irrespective of the phasing method.

## Methods

### PhaseME prerequisites

PhaseME requires phased VCF files as input, which need to follow the VCF standards 4.1 or newer [30]. For processing, PhaseME requires tags (PS and GT) to identify the phase blocks and genotypes such as 0|1 or 1|0 to indicate the haplotypes per SNV. PhaseME is written in Python3 and requires the Numpy package.

To exploit population information and obtain the linkage information, we used Shapeit2 (version v2r900) [31] based on the 1000 Genome dataset [25]. Because the Shapeit2 package needs phased data per chromosome, we split the input VCF file into 22 VCF files corresponding to 22 chromosomes. We also removed non-genotyped variants from each VCF. Using the “-check” subprogram of Shapeit2 with “–input-vcf” option, we report the missing variants in the reference panel, which is then excluded using the “–exclude-snp” option of Shapeit2. Then we generate a haplotype graph, which is a compact format of population information using the “–output-graph” option accompanying the genetic map with “-M” and phased haplotype, legend, and sample names with “–input-ref” options. Then using the “-convert” subprogram of Shapeit2, we sample the haplotype graph and generate haplotype samples (default 500 times). Subsequently, haplotype samples are used to generate a pair list inspired by Bansal [24]. Each element of the list contains the positions of 2 SNVs and the relation of their phased GT. If the phasing of 2 variants in 90% of samples are identical (or opposite), we will report them in the pair list.

### Definition of quality criteria

Most of the measurements reported by PhaseME are self-explained such as the number of heterozygous SNVs, phased SNVs, N50 phasing, and non-phased heterozygous SNVs to give insights into the phasing performance. Nevertheless, a few metrics exist that we describe here in detail.

For calculating the mean phase block quality, we averaged the ratio of the number of non-conflicting SNVs over the all SNVs along each chromosome based on the linkage information. Phase rate is calculated by dividing the summation of phase block length by the difference in the position of last and first SNVs of the chromosome.

### Data description

The raw reads were obtained from GIAB ftp [32] (ONT [33], Illumina [34], PacBio Hi-Fi [35], and PacBio CLR [36]). The ONT and PacBio reads were aligned using NGMLR [37] to the human reference genome (HG19). Subsequently, we identified SNVs using the Clair2 package [38]. For Illumina, we used xatlas for calling SNVs [39]. We used WhatsHap [23] to phase the SNVs based on sequencing reads. The phased data of 10x Genomics were downloaded from GIAB ftp [40].

We downloaded 1000 Genome data phase 3, including 2,504 samples [41]. The population information was downloaded from [42] including phased haplotype in IMPUTE format (that consists of 0 s [reference allele] and 1 s [alternate allele]), the legend file in IMPUTE format including the genomic position of variants, the reference and alternate allele in base, the allele frequency in populations, and the genetic map file in IMPUTE format of physical positions in NCBI b37 coordinates. The membership of each sample is reported in the “1000GP_Phase3.sample” file.

### Benchmarking of variant calls

We downloaded the GIAB gold-standard call set of HG002 from [43]. To compare the called SNVs with the gold standard, we use the vcfeval subprogram of rtg tools [44]. We reported the accuracy on the calls without filtering based on the quality values.

### Benchmarking of phasing data

Here we used parental information to benchmark the results of PhaseME. We have downloaded the high-confidence parental call set from GIAB ftp [45] and [46]. For each technology, we generate the phased SNV VCF (see above) for the HG002 son. To generate the parental set, we first combine all 3 call sets of the son (being the one to be benchmarked), mother, and father using bcftools merge [30]. Then by considering the heterozygous SNVs, we generate a phasing set for the son using in-house Python code. Given the overlap between a heterozygous SNV of the son and only the father, we report the phasing as 0|1. If overlaps exclusively with the mother, 1|0 is reported. The output VCF file is used for evaluation of the individual phasing method to calculate the Hamming error rate, the number of short and long switches using an in-house Python code provided on our GitHub page.

The Hamming error rate is defined as the number of the individual's phasings that are different from the true phasing divided by the number of phased heterozygous variants. A switch is defined by comparing the phased VCF with true. For each phase block we first compute the number of agreeing and disagreeing phasing information. If the majority of SNV are disagreeing, we need to consider the possibility that the phase block is reported the other way around and thus invert the result. Subsequently the remaining mismatching phase genotypes represent errors in the phasing, i.e., switch errors, if there are multiple in a row. The reported results are averaged over phase blocks.

## Availability of Supporting Source Code and Requirements

Project name: PhaseME

Project home page: https://github.com/smajidian/phaseme

Operating system: Linux

Programming language: Python

Other requirements: Python 3.6 or higher

License: MIT License

Biotools identifier: phaseme (https://bio.tools/phaseme)

RRID:SCR_018739

## Availability of Supporting Data and Materials

The data set supporting the results of this article is available in the GigaDB repository [47].

## Additional Files

**Supplementary Table 1**. Number of long switches for different thresholds using precomputed pair lists.

**Supplementary Table 2**. Number of short switches for different thresholds using precomputed pair lists.

**Supplementary Table 3**. Benchmarking of variant calls of chromosome 9 for 5 technologies.

**Supplementary Figure 1**. Number of long switch errors vs the length of phase blocks in which each point is a chromosome for 4 technologies. No correlation between the number of long switch errors and the length of phase blocks has been observed based on long read–based phasing.

**Supplementary Figure 2**. Results of running PhaseME in parental mode. The number of long switches is decreased to zero without affecting the phase block length.

giaa078_GIGA-D-20-00099_Original_Submission

giaa078_GIGA-D-20-00099_Revision_1

giaa078_GIGA-D-20-00099_Revision_2

giaa078_Response_to_Reviewer_Comments_Original_Submission

giaa078_Response_to_Reviewer_Comments_Revision_1

giaa078_Reviewer_1_Report_Original_Submission Arang Rhie -- 5/5/2020 Reviewed

giaa078_Reviewer_1_Report_Revision_1 Arang Rhie -- 6/16/2020 Reviewed

giaa078_Reviewer_2_Report_Original_Submission Cinta Pegueroles -- 5/9/2020 Reviewed

giaa078_Reviewer_2_Report_Revision_1 Cinta Pegueroles -- 6/2/2020 Reviewed

giaa078_Supplemental_Tables

## Abbreviations

bp: base pair; CLR: continuous long reads; CPU: central processing unit; GIAB: Genome in a Bottle; kb: kilobase pairs; Mb: megabase pairs; NCBI: National Center for Biotechnology Information; ONT: Oxford Nanopore Technologies; PacBio: Pacific Biosciences; SIH: single individual haplotyping; SNP: single-nucleotide polymorphism; SNV: single-nucleotide variation; SV: structural variation; TPMT: thiopurine S-methyltransferase; VCF: Variant Call Format.

## Competing Interests

F.J.S. has participated in PacBio and Oxford Nanopore sponsored meetings over the past few years and has received travel reimbursement for presenting at these events. F.J.S. also received the SMRT PacBio sequencing grant in 2018. S.M. declares that he has no competing interests.

## Funding

This work was supported in part by the US National Institutes of Health (UM1 HG008898).

## Authors' Contributions

The project was conceived by F.J.S. Methods were designed by both authors. Algorithm code was implemented by S.M. Both authors wrote the manuscript. F.J.S. supervised the project.

## References

[1] Sedlazeck FJ , Lee H, Darby CA, et al. Piercing the dark matter: bioinformatics of long-range sequencing and mapping. Nat Rev Genet. 2018;19:329–46. 29599501 10.1038/s41576-018-0003-4

[2] Browning SR , Browning BL. Haplotype phasing: existing methods and new developments. Nat Rev Genet. 2011;12:703–14. 21921926 10.1038/nrg3054 PMC3217888

[3] Snyder MW , Adey A, Kitzman JO, et al. Haplotype-resolved genome sequencing: experimental methods and applications. Nat Rev Genet. 2015:344–58. 25948246 10.1038/nrg3903

[4] Zhang X , Wu R, Wang Y, et al. Unzipping haplotypes in diploid and polyploid genomes. Comput Struct Biotechnol J. 2020;18:66–72. 31908732 10.1016/j.csbj.2019.11.011 PMC6938933

[5] Choi Y , Chan AP, Kirkness E, et al. Comparison of phasing strategies for whole human genomes. PLoS Genet. 2018;14:e1007308. 29621242 10.1371/journal.pgen.1007308 PMC5903673

[6] Beck CR , Carvalho CMB, Akdemir ZC, et al. Megabase length hypermutation accompanies human structural variation at 17p11.2. Cell. 2019;176:1310–24.e10. 30827684 10.1016/j.cell.2019.01.045 PMC6438178

[7] Yang H , Spitz MR, Stewart DJ, et al. ATM sequence variants associate with susceptibility to non-small cell lung cancer. Int J Cancer. 2007;121:2254–9. 17582598 10.1002/ijc.22918 PMC3477817

[8] Barroso E , Milne RL, Fernández LP, et al. FANCD2 associated with sporadic breast cancer risk. Carcinogenesis. 2006;27:1930–7. 16679306 10.1093/carcin/bgl062

[9] Pelletier C , Speed WC, Paranjape T, et al. RareBRCA1haplotypes including 3′UTR SNPs associated with breast cancer risk. Cell Cycle. 2011;10:90–9. 21191178 10.4161/cc.10.1.14359 PMC3048078

[10] Leija-Salazar M , Sedlazeck FJ, Toffoli M, et al. Evaluation of the detection of GBA missense mutations and other variants using the Oxford Nanopore MinION. Mol Genet Genomic Med. 2019;7:e564. 30637984 10.1002/mgg3.564 PMC6418358

[11] Tewhey R , Bansal V, Torkamani A, et al. The importance of phase information for human genomics. Nat Rev Genet. 2011;12:215–23. 21301473 10.1038/nrg2950 PMC3753045

[12] Liu P-Y , Zhang Y-Y, Lu Y, et al. A survey of haplotype variants at several disease candidate genes: the importance of rare variants for complex diseases. J Med Genet. 2005;42:221–7. 15744035 10.1136/jmg.2004.024752 PMC1736011

[13] Appell ML , Berg J, Duley J, et al. Nomenclature for alleles of the thiopurine methyltransferase gene. Pharmacogenet Genomics. 2013;23:242–8. 23407052 10.1097/FPC.0b013e32835f1cc0 PMC3727893

[14] Almoguera B , Vazquez L, Connolly JJ, et al. Imputation of TPMT defective alleles for the identification of patients with high-risk phenotypes. Front Genet. 2014;5:96. 24860591 10.3389/fgene.2014.00096 PMC4026736

[15] Kurzawski M , Dziewanowski K, Ciechanowski K, et al. Severe azathioprine-induced myelotoxicity in a kidney transplant patient with thiopurine S-methyltransferase-deficient genotype (TPMT*3A/*3C). Transpl Int. 2005;18:623–5. 15819814 10.1111/j.1432-2277.2005.00095.x

[16] Relling MV , Schwab M, Whirl-Carrillo M, et al. Clinical Pharmacogenetics Implementation Consortium Guideline for thiopurine dosing based on TPMT and NUDT15 genotypes: 2018 update. Clin Pharmacol Ther. 2019;105:1095–105. 30447069 10.1002/cpt.1304 PMC6576267

[17] Huang M , Tu J, Lu Z. Recent advances in experimental whole genome haplotyping methods. Int J Mol Sci. 2017;18:1944. 28891974 10.3390/ijms18091944 PMC5618593

[18] Ma L , Xiao Y, Huang H, et al. Direct determination of molecular haplotypes by chromosome microdissection. Nat Methods. 2010;7:299–301. 20305652 10.1038/nmeth.1443 PMC2871314

[19] Browning SR , Browning BL. Rapid and accurate haplotype phasing and missing-data inference for whole-genome association studies by use of localized haplotype clustering. Am J Hum Genet. 2007;81:1084–97. 17924348 10.1086/521987 PMC2265661

[20] Browning BL , Browning SR. A fast, powerful method for detecting identity by descent. Am J Hum Genet. 2011;88:173–82. 21310274 10.1016/j.ajhg.2011.01.010 PMC3035716

[21] Garg S , Martin M, Marschall T. Read-based phasing of related individuals. Bioinformatics. 2016;32:i234–42. 27307622 10.1093/bioinformatics/btw276 PMC4908360

[22] Edge P , Bafna V, Bansal V. HapCUT2: robust and accurate haplotype assembly for diverse sequencing technologies. Genome Res. 2017;27:801–12. 27940952 10.1101/gr.213462.116 PMC5411775

[23] Martin M , Patterson M, Garg S, et al. WhatsHap: fast and accurate read-based phasing. bioRxiv. 2016, doi:10.1101/085050.

[24] Bansal V . Integrating read-based and population-based phasing for dense and accurate haplotyping of individual genomes. Bioinformatics. 2019;35:i242–8. 31510646 10.1093/bioinformatics/btz329 PMC6612846

[25] Zook JM , Catoe D, McDaniel J, et al. Extensive sequencing of seven human genomes to characterize benchmark reference materials. Sci Data. 2016;3:160025. 27271295 10.1038/sdata.2016.25 PMC4896128

[26] Zook JM , Hansen NF, Olson ND, et al. A robust benchmark for germline structural variant detection. bioRxiv. 2019, doi:10.1101/664623.

[27] Wenger AM , Peluso P, Rowell WJ, et al. Accurate circular consensus long-read sequencing improves variant detection and assembly of a human genome. Nat Biotechnol. 2019;37:1155–62. 31406327 10.1038/s41587-019-0217-9 PMC6776680

[28] Zook JM , Chapman B, Wang J, et al. Integrating human sequence data sets provides a resource of benchmark SNP and indel genotype calls. Nat Biotechnol. 2014;32:246–51. 24531798 10.1038/nbt.2835

[29] Porubsky D , Garg S, Sanders AD, et al. Dense and accurate whole-chromosome haplotyping of individual genomes. Nat Commun. 2017;8:1293. 29101320 10.1038/s41467-017-01389-4 PMC5670131

[30] Li H . A statistical framework for SNP calling, mutation discovery, association mapping and population genetical parameter estimation from sequencing data. Bioinformatics. 2011;27:2987–93. 21903627 10.1093/bioinformatics/btr509 PMC3198575

[31] Delaneau O , Zagury J-F, Marchini J. Improved whole-chromosome phasing for disease and population genetic studies. Nat Methods. 2013;10:5–6. 23269371 10.1038/nmeth.2307

[32] GIAB: GIAB project site. https://jimb.stanford.edu/giab/. Accessed on 14th July 2020.

[33] GIAB: GIAB ONT reads of HG002. ftp://ftp-trace.ncbi.nlm.nih.gov/giab/ftp/data/AshkenazimTrio/HG002_NA24385_son/UCSC_Ultralong_OxfordNanopore_Promethion/. Accessed on 14th July 2020.

[34] GIAB: GIAB Illumina reads of HG002. ftp://ftp-trace.ncbi.nlm.nih.gov/giab/ftp/data/AshkenazimTrio/HG002_NA24385_son/NIST_Illumina_2x250bps/novoalign_bams/. Accessed on 14th July 2020.

[35] GIAB: GIAB Hi-Fi reads of HG002. ftp://ftp-trace.ncbi.nlm.nih.gov/giab/ftp/data/AshkenazimTrio/HG002_NA24385_son/PacBio_CCS_15kb/. Accessed on 14th July 2020.

[36] GIAB: GIAB CLR reads of HG002. ftp://ftp-trace.ncbi.nlm.nih.gov/giab/ftp/data/AshkenazimTrio/HG002_NA24385_son/PacBio_MtSinai_NIST/. Accessed on 14th July 2020.

[37] Sedlazeck FJ , Rescheneder P, Smolka M, et al. Accurate detection of complex structural variations using single-molecule sequencing. Nat Methods. 2018;15:461–8. 29713083 10.1038/s41592-018-0001-7 PMC5990442

[38] Luo R , Wong C-L, Wong Y-S, et al. Exploring the limit of using a deep neural network on pileup data for germline variant calling. Nat Mach Intell. 2020;2:220–7.

[39] Farek J , Hughes D, Mansfield A, et al. xAtlas: Scalable small variant calling across heterogeneous next-generation sequencing experiments. bioRxiv. 2020, doi:10.1101/295071. PMC9841152 36644891

[40] GIAB: 10x Genomics-based phased data of HG002. ftp://ftp-trace.ncbi.nlm.nih.gov/giab/ftp/data/AshkenazimTrio/analysis/10XGenomics_ChromiumGenome_LongRanger2.2_Supernova2.0.1_04122018/GRCh37/NA24385_LongRanger_snpindel.vcf.gz. Accessed on 14th July 2020.

[41] 1000 Genomes Project Consortium, Auton A, Brooks LD, Durbin RM, et al. A global reference for human genetic variation. Nature. 2015;526:68–74. 26432245 10.1038/nature15393 PMC4750478

[42] 1000 Genomes haplotypes . https://mathgen.stats.ox.ac.uk/impute/1000GP_Phase3.tgz.Accessed on 14th July 2020.

[43] GIAB: GIAB SNV calls gold standard of HG002. ftp://ftp-trace.ncbi.nlm.nih.gov/giab/ftp/release/AshkenazimTrio/HG002_NA24385_son//NISTv3.3.2/GRCh37/HG002_GRCh37_GIAB_highconf_CG-IllFB-IllGATKHC-Ion-10X-SOLID_CHROM1-22_v.3.3.2_highconf_triophased.vcf.gz. Accessed on 14th July 2020.

[44] Cleary JG , Braithwaite R, Gaastra K, et al., Comparing variant call files for performance benchmarking of next-generation sequencing variant calling pipelines, bioRxiv, 2015, doi:10.1101/023754.

[45] GIAB: GIAB SNV calls gold standard of HG003. ftp://ftp-trace.ncbi.nlm.nih.gov/giab/ftp/release/AshkenazimTrio/HG003_NA24149_father/NISTv3.3.2/GRCh37/HG003_GRCh37_GIAB_highconf_CG-IllFB-IllGATKHC-Ion-10X_CHROM1-22_v.3.3.2_highconf.vcf.gz. Accessed on 14th July 2020.

[46] GIAB: GIAB SNV calls gold standard of HG004. ftp://ftp-trace.ncbi.nlm.nih.gov/giab/ftp/release/AshkenazimTrio/HG004_NA24143_mother/NISTv3.3.2/GRCh37/HG004_GRCh37_GIAB_highconf_CG-IllFB-IllGATKHC-Ion-10X_CHROM1-22_v.3.3.2_highconf.vcf.gz. Accessed on 14th July 2020.

[47] Majidian S , Sedlazeck FJ. Supporting data for “PhaseME: automatic rapid assessment of phasing quality and phasing improvement.”. GigaScience Database. 2020. 10.5524/100768. PMC7379178 32706368

