# Supplementary material for: PhaseME: Automatic rapid assessment of phasing quality and phasing improvement
Source: Gigascience. 2020 Jul 24;9(7):giaa078. doi: 10.1093/gigascience/giaa078 (PMC7379178; doi:10.1093/gigascience/giaa078)
Supplement: giaa078_GIGA-D-20-00099_Original_Submission [file giaa078_giga-d-20-00099_original_submission.pdf]

GigaScience

PhaseME: automatic rapid assessment of phasing quality and phasing improvement

--Manuscript Draft--

**Manuscript Number:**	GIGA-D-20-00099
**Full Title:**	PhaseME: automatic rapid assessment of phasing quality and phasing improvement
**Article Type:**	Technical Note
**Funding Information:**	National Institutes of Health (UM1 HG008898) — Not applicable
**Abstract:**	Background<br><br>The detection of what mutations are occurring on the same DNA molecule is essential to predict their consequences. This can be achieved by phasing the genomic variations. Nevertheless, state-of-the-art haplotype phasing is currently a black box in which the accuracy and quality of the reconstructed haplotypes are hard to assess.<br><br>Findings<br><br>Here we present PhaseME, a versatile method to provide insights into and improvement of sample phasing results based on linkage data. We showcase the performance and the importance of PhaseME by comparing phasing information obtained from Pacific Biosciences (PacBio) including both CLR (continuous long reads) and HiFi (high-quality consensus reads), Oxford Nanopore Technologies (ONT) and 10Xgenomics sequencing technologies. We found that 10Xgenomics and ONT phasing can be significantly improved while retaining a high N50 and completeness. PhaseME generates reports and summary plots to provide insights into phasing performance and correctness. We observed unique phasing issues for each of the sequencing technologies, highlighting the necessity of quality assessments. PhaseME is able to decrease the Hamming error rate significantly by 26.2% averaged across all four technologies. Additionally, a significant improvement is obtained in the number of long switch errors especially for HiFi 54.6% with only a 5% decrease in phase block N50.<br><br>Conclusions<br><br>PhaseME is a universal method to assess the phasing quality and accuracy and improves the quality of phasing using linkage information. The package is freely available at https://github.com/smajidian/phaseme .
**Corresponding Author:**	Fritz Sedlazeck<br><br>UNITED STATES
**Corresponding Author Secondary Information:**	
**Corresponding Author's Institution:**	
**Corresponding Author's Secondary Institution:**	
**First Author:**	Sina Majidian
**First Author Secondary Information:**	
**Order of Authors:**	Sina Majidian
	Fritz Sedlazeck
**Order of Authors Secondary Information:**	
**Additional Information:**	
**Question**	**Response**

Powered by Editorial Manager® and ProduXion Manager® from Aries Systems Corporation

Are you submitting this manuscript to a special series or article collection?	No
**Experimental design and statistics**<br><br>Full details of the experimental design and statistical methods used should be given in the Methods section, as detailed in our Minimum Standards Reporting Checklist. Information essential to interpreting the data presented should be made available in the figure legends.<br><br>Have you included all the information requested in your manuscript?	Yes
**Resources**<br><br>A description of all resources used, including antibodies, cell lines, animals and software tools, with enough information to allow them to be uniquely identified, should be included in the Methods section. Authors are strongly encouraged to cite Research Resource Identifiers (RRIDs) for antibodies, model organisms and tools, where possible.<br><br>Have you included the information requested as detailed in our Minimum Standards Reporting Checklist?	Yes
**Availability of data and materials**<br><br>All datasets and code on which the conclusions of the paper rely must be either included in your submission or deposited in publicly available repositories (where available and ethically appropriate), referencing such data using a unique identifier in the references and in the "Availability of Data and Materials" section of your manuscript.<br><br>Have you have met the above requirement as detailed in our Minimum Standards Reporting Checklist?	Yes

Manuscript

Click here to download Manuscript PhaseME_manuscript.docx

PhaseME: automatic rapid assessment of phasing quality and phasing improvement

Sina Majidian[1], Fritz J. Sedlazeck[2]*

[1]School of Electrical Engineering, Iran University of Science & Technology, Narmak, Tehran, Iran. 1684613114

[2]Human Genome Sequencing Center, Baylor College of Medicine, 1 Baylor Plaza, Houston, TX 77030, USA

*Correspondence: fritz.sedlazeck@bcm.edu

Abstract

Background: The detection of what mutations are occurring on the same DNA molecule is essential to predict their consequences. This can be achieved by phasing the genomic variations. Nevertheless, state-of-the-art haplotype phasing is currently a black box in which the accuracy and quality of the reconstructed haplotypes are hard to assess.

Findings: Here we present PhaseME, a versatile method to provide insights into and improvement of sample phasing results based on linkage data. We showcase the performance and the importance of PhaseME by comparing phasing information obtained from Pacific Biosciences (PacBio) including both CLR (continuous long reads) and HiFi (high-quality consensus reads), Oxford Nanopore Technologies (ONT) and 10Xgenomics sequencing technologies. We found that 10Xgenomics and ONT phasing can be significantly improved while retaining a high N50 and completeness. PhaseME generates reports and summary plots

to provide insights into phasing performance and correctness. We observed unique phasing issues for each of the sequencing technologies, highlighting the necessity of quality assessments. PhaseME is able to decrease the Hamming error rate significantly by 26.2% averaged across all four technologies. Additionally, a significant improvement is obtained in the number of long switch errors especially for HiFi 54.6% with only a 5% decrease in phase block N50.

Conclusions: PhaseME is a universal method to assess the phasing quality and accuracy and improves the quality of phasing using linkage information. The package is freely available at https://github.com/smajidian/phaseme.

Keywords:

DNA sequencing; haplotype phasing; bioinformatics; quality control.

Findings

Humans as well as many other organisms have a diploid genome, meaning that there are two homologous copies of every somatic chromosome inherited from mother and father. These copies include genomic variation including Single Nucleotide Variation (SNV) and Structural Variation (SV) [1]. Each variation represents a difference in a nucleotide(s) unique to each of the chromosome copies also called haplotypes [2]. Thus, haplotypes represent the individual copy of each genomic element and need to be studied independently to investigate the impact of variations.

Haplotype phase information is essential to understand where a mutation occurs, predict their interactions (*i.e.* if two SNVs are on the same DNA molecule) and their potential impact on genes and their expression and thus phenotypes. This is important for multiple applications

and organisms. For humans, phasing plays an important role in *e.g*. Mendelian diseases [3], cancer genomics [4–6], neurological diseases [7], genetic research and other medical applications [8,9]. As an example, compound heterozygosity shows the importance of phase information for relating genotype to phenotype. Numerous examples of disorders influenced by compound heterozygosity are known [8]. For example, Thiopurine S-methyltransferase (TPMT) is a 27 kbp gene located on chromosome 6p22.3. It is translated into an enzyme catalyzing S-methylation of thiopurine drugs [10]. These drugs are used as chemotherapeutic and immunosuppressant agents in lymphoid malignancies, leukemia and inflammatory bowel disease. The enzyme activity is controlled by the genetic polymorphism of the gene. There are two SNPs (rs1800460 and rs1142345) where either are known to impact the activity of TPMT and cause missense changes[11]. Thus, it is important to know if both SNPs exist, and if so if they co-occur on the same haplotype (cis) leading to an inactivation of TPMT or not (trans). Patients with low TPMT activity are at higher risk of life-threatening severe myelosuppression and hematopoietic toxicity when treated with conventional doses of mercaptopurine (or azathioprine) [12-13].

Currently, we distinguish four different approaches to obtain phasing information [14]:

1. Wet-lab based phasing is based on mainly two different methods: encapsulation and 3D structure capture [15]. One method is to extract chromosomes when cells are in metaphase and then microdissect them into subsets [16].
2. Population-based phasing, which utilizes linkage information derived from hundreds to thousands of individuals [17]. However, this approach misses rare and *de novo* variants [18].
3. Parental methods [19] have the advantage to phase entire genomes but lack the ability to phase *de novo* mutations (*i.e.* important in many Mendelian diseases). Furthermore, it requires sequencing of the parents, which is more expensive and often not applicable.

4. Single individual haplotyping (SIH), *or* haplotype assembly, is the most comprehensive approach; since it includes *de novo* mutations and rare variations, but often suffers from fragmentation [1]. HapCut2 [20] and WhatsHap [21] are the two most commonly used algorithms for this approach capable of utilizing Illumina, PacBio, Oxford Nanopore, 10Xgenomics and HiC information. In short, these approaches rely on aligned reads to a reference genome from which the SNVs and their genotypes are inferred. Subsequently, the SIH methods cluster the reads along the heterozygous SNV into two groups corresponding to the two haplotypes. The resulting VCF file reports SNVs with their phasing information, which includes the assignment of each SNV to a phase block. It is important to note that the relationship between individual phase blocks remains undetermined [14].

Based on the above information SIH methods are important and necessary for a better understanding of the genome at hand. However, it remains tedious to impossible to assess the accuracy and even the performance of individual samples. Generally, all of them may suffer from inaccurate results, which includes errors in the grouping of reads leading to incorrectly assigned SNVs (flip errors) or inaccurately joined haplotypes (switch errors). Currently phasing is often evaluated based solely on its phasing length e.g. N50. Phasing N50 is the minimum phase block length, where the sum of its phase blocks with all larger phase blocks span at least 50% of the total phase length. Similar to assembly methods however, N50 does not represent the accuracy or quality of a result. In addition, most phasing methods do not provide a quality score to assess the reliability of the phasing itself. Making it near impossible for users to assess the quality/correctness of their results.

To solve these problems and limitations we developed PhaseME, a method to automatically estimate the quality of the SIH results and report multiple statistics to enable a deeper understanding for a broad range of users. This is done based on population data to assess the accuracy of common variations across the individual phasing information. Furthermore,

PhaseME can detect phasing errors and highlight their locations. If desired, PhaseME continues to correct the detected phasing errors and generates a report of the improvement and impact of these changes. Thus, PhaseME is unique in its usability and application as it provides more insights into the phasing accuracy per sample. To the best of our knowledge, there exists only one other highly specialized tool, which requires population information together with Hi-C data to correct SIH phasing and does not provide insights in the phasing results [22]. In the following, we describe the features of PhaseME and its applications for different technologies based on SIH phasing for HG002. We assess the performance of PhaseME based on parental phasing information from GIAB [23] across four different sequencing technologies .

PhaseME

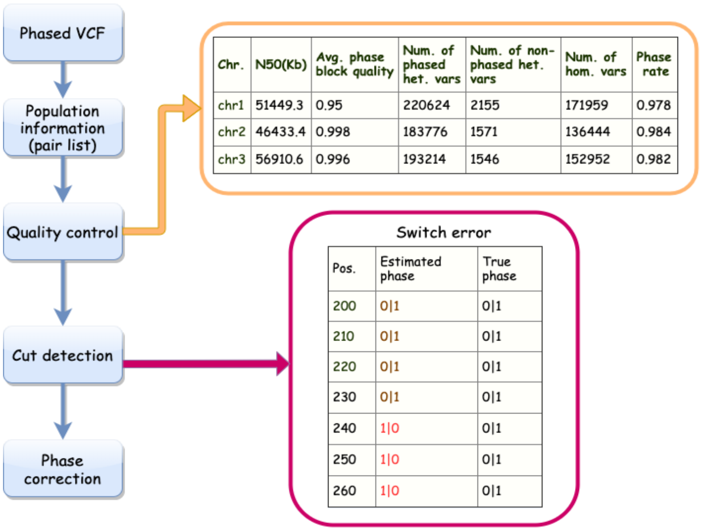

***Figure 1*:.** *Summary of PhaseME. PhaseME consists of three steps: extracting population information, quality control, and phase error correction.*

PhaseME is a novel method to assess the quality of phasing results and provide a quality control report. It also reduces phasing errors by exploiting population information. **Figure 1** gives an overview of the three main steps of PhaseME. First, PhaseME requires the phased SNVs VCF file for an individual obtained from a SIH method, which is compared to precomputed linkage information that is available per ethnicity (see the **Methods Section** for details).

Second, PhaseME reports an in-depth quality assessment report of the phasing result to provide a detailed overview. PhaseME calculates the quality ratio across phase blocks based on the previously obtained linkage information. Here, for each phase block, we compute the ratio of SNVs with non-conflicting to conflicting linkage information and report the average per chromosome. Thus, 0 represents the lowest accuracy, while 1 indicates that everything is supported by the linkage information and more likely correct. PhaseME further reports the N50 of phase block length in kbp, the number of phased and non-phased heterozygous and homozygous variants, average phase block quality and phase rate (see the Quality control and Methods Section for details).

Third, PhaseME corrects the previously identified SNVs that are in conflict with the linkage information. We distinguish small (2-20bp) and large (21bp+) switch errors that represent a stretch of incorrectly phased SNV and thus PhaseME splits the existing phase block into two at the first conflicting SNV (see the section on error correction below for details).

Quality control

As highlighted above, it is essential to obtain insights into the phasing quality. PhaseME is designed with this as its main application and to provide an easy to understand and comprehensive quality report across the phasing results. We outline the provided summaries below based on SIH results for HG002 from GIAB [24] (see the **Methods Section**).

We evaluate the accuracy of SNV phasing across ONT, PacBio CLR, PacBio HiFi [25] and 10Xgenomics. For each SNV, we consider the linkage information obtained from the 1000 Genomes Project of the population data if it is in conflict (*i.e.* mismatched) or in agreement (*i.e.* matched). Thus the higher the number of matched SNVs, the better is the phase quality of the phase block.

To inspect the haplotype length, PhaseME reports: i) N50 of phase block length in kbp, which highlights the overall length of the phasing, ii) number of phased and non-phased heterozygous variants, which illustrates the completeness of the phasing, iv) the phase rate to indicate the proportion of phased regions for each chromosome, v) the average phase block quality to indicate the agreement with the linkage information and vi) the number of homozygous variants. The detailed definition of each criterion is provided in the **Methods Section**. Based on this report, one can determine the phasing quality for each chromosome and phase block of the sample at hand. Each of these statistics is automatically generated and provided by PhaseME in the QC report file.

As expected we observed a high N50 phase block length for 10Xgenomics (10.9Mb), but interestingly even higher for ONT (15.6Mbp) compared to PacBio CLR (369.3kbp) or PacBio HiFi (314.3kbp) (**Figure 2A**). One likely reason is the longer read lengths of the technologies compared to PacBio. Interestingly PhaseME highlights a higher number of heterozygous SNVs for 10Xgenomics (2,890,988) followed by ONT (2,807,291), whereas for PacBio we observed a lower number of heterozygous SNVs, CLR (2,520,418) and HiFi (2,418,009) (see **Figure 2B**). For 10Xgenomics the rate of non-phased heterozygous SNVs is also high in contrast to ONT (**Figure 2C**). The results of PhaseME show that the average phase block quality was the highest for HiFi (0.994) followed by 10Xgenomics (0.985), ONT (0.983) and CLR (0.978). We observed a lower phase quality for chromosome 9 when using ONT and 10Xgenomics data (see **Figure 2D**). However, we did not observe that this affects the same regions.

It is interesting to note that the PacBio CLR data shows a lower N50 and a lower phase accuracy compared to ONT. However, this might be explained by the fact that CLR data is from 2015 and was generated with the RSII instrument for GIAB [23]. Another important observation is that the ONT data has the largest N50 phase length but is also quite accurate only 0.11 behind the HiFi reads which was the most accurate, but has an almost 60 times

reduced N50. Another important metric is the phasing completeness, which represents the fraction of regions phased along the genome. **Figure 2E** shows the results based on the PhaseME report across the technologies. 10Xgenomics (0.947) and ONT (0.949) have the highest ratio of phased heterozygous SNV. This is followed by CLR (0.75) and HiFi (0.69) probably strongly related to the molecule/read size and their higher number of heterozygous SNV. 10Xgenomics (22.7%) showed by far the highest ratio of non-phased heterozygous SNV followed by ONT (1.1%), whereas for PacBio we observed a lower ratio CLR (0.34%) and HiFi (0.32%) (see **Figure 2C**).

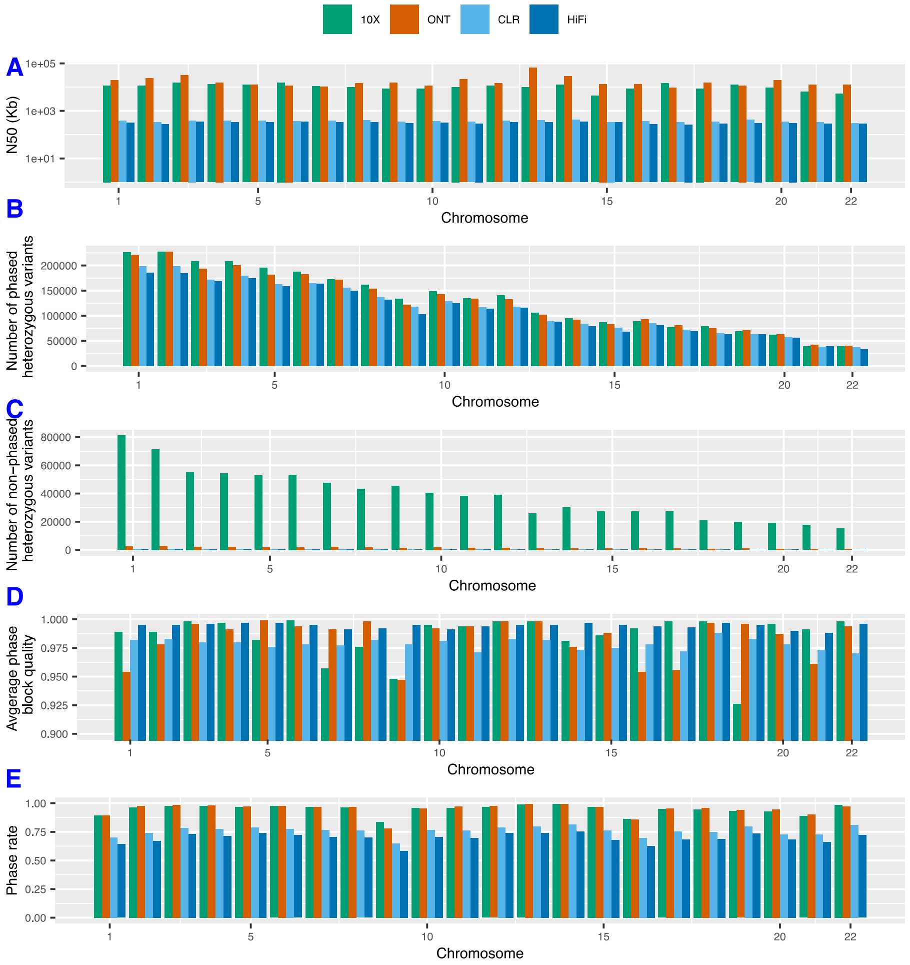

Figure 2: Phasing Quality Overview. *Quality of reconstructed haplotypes for HG002 by WhatsHap across the different technologies: 10Xgenomics (green), ONT (orange), PacBio CLR (light blue) and PacBio HiFi (blue). A) N50 of phase block length in kbp (in logarithmic scale). B) Number of phased heterozygous variants for each technology highlighting a higher number of heterozygous SNV in general for 10Xgenomics and ONT. C) Comparison of the non-phased heterozygous variations along the chromosomes. D) Average phase block quality and E) Phase rate based on the four technologies*

Phasing error correction

Phasing errors lead to the misassignment of mutations to the wrong haplotype. PhaseME aims to correct larger switch errors as the linkage data does not provide sufficient resolution to correct single flip errors. To detect switch errors, PhaseME considers mismatches indicated by upstream SNVs to improve the signal. PhaseME requires a minimum of two mismatches and considers the ratio between matching and mismatching linkage information for the SNVs. If this ratio (matches/mismatches) < 1 (by default) then PhaseME breaks the section in two separate phase blocks with the first conflicting SNV as the start of the new phase block.

We benchmark the error correction ability of PhaseME across different sequencing technologies (ONT, PacBio HiFi, PacBio CLR, 10Xgenomics) based on a male Ashkenazi proband, HG002 (NA24385) from the Genome in a Bottle (GIAB) repository (see the **Methods Section**), and compared the results to the parental phasing information based on Illumina SNVs [23]. Note that HG002 is not included in the population dataset (1000G data) that was used to obtain the linkage information.

We also consider the Hamming error rate [26] to evaluate the phasing, which shows the fraction of incorrectly phased over the total number of phased heterozygous variants (see the **Methods Section**). ONT (0.203) has the highest Hamming error rate followed by 10Xgenomics (0.073) PacBio CLR (0.0389), and PacBio HiFi (0.0098) with the smallest Hamming error rate. This seems a bit contradictory to our previous results where ONT had a high average phasing quality (**Figure 2D)**. We did not observe a correlation between the number of long switch errors and the length of phase blocks (Spearman's correlation test p-value = 0.14 (ONT), 0.19 (10Xgenomics), 0.69 (PacBio CLR) and 0.66 (PacBio HiFi)) and thus explains the different results (see **Supplementary Figure 1**). This is likely due to higher switch error rates in ONT (780) vs the other technologies PacBio CLR (497), PacBio HiFi (230) and 10Xgenomics (50). Clearly, these errors are also related to the overall size of phase blocks where ONT (15.6Mbp) and 10Xgenomics (10.9Mb) had the longest N50. For short 2-20bp switches, we observed the highest number of errors for CLR (1,452) followed by 10Xgenomics (1,356), ONT (698), and PacBio HiFi (455).

PhaseME overall reduced the phasing errors based on the linkage information. **Figure 3** shows the results of improving the phasing and impact on phase block length for PhaseME. For the Hamming error rate, we observed on average a 26.2% reduction in errors across the technologies. For ONT, the reduction was the highest of 34.3% followed by 10Xgenomics (25.1%), PacBio HiFi (24.5%) and PacBio CLR (20.8%). **Figure 3** shows the improvement of

long switch errors (20 SNP+). Here PhaseME reduced the error for the ONT data set from 780 down to 647 (17.0%). This resulted in a reduced N50 from 15.6Mbp down to 6.3Mbp. For CLR, PhaseME decreased the long switch errors by 46.0%, which led to a decrease of phasing N50 from 369.3kbp to 339.1kbp. For 10Xgenomics we could improve the phasing by 23.5% with a reduced N50 from 10.9Mb to 5.9Mbp. The number of long switch errors for HiFi was reduced by 54.6% (see **Figure 3**) which leads to a reduced N50 from 314.3kbp to 300.3kbp. Next, we evaluated PhaseME for short switches (2-20bp). Here, the linkage data does not provide the resolution to improve most of them. Thus, the number of short switches (with the length of <20) is decreased for CLR (22.4%), HiFi (18.6%) and 10Xgenomics (1.5%). However, our comparisons to the parents indicated that for ONT we actually introduced 6.6% of short switch errors. Thus, we provide parameters to adjust PhaseME.

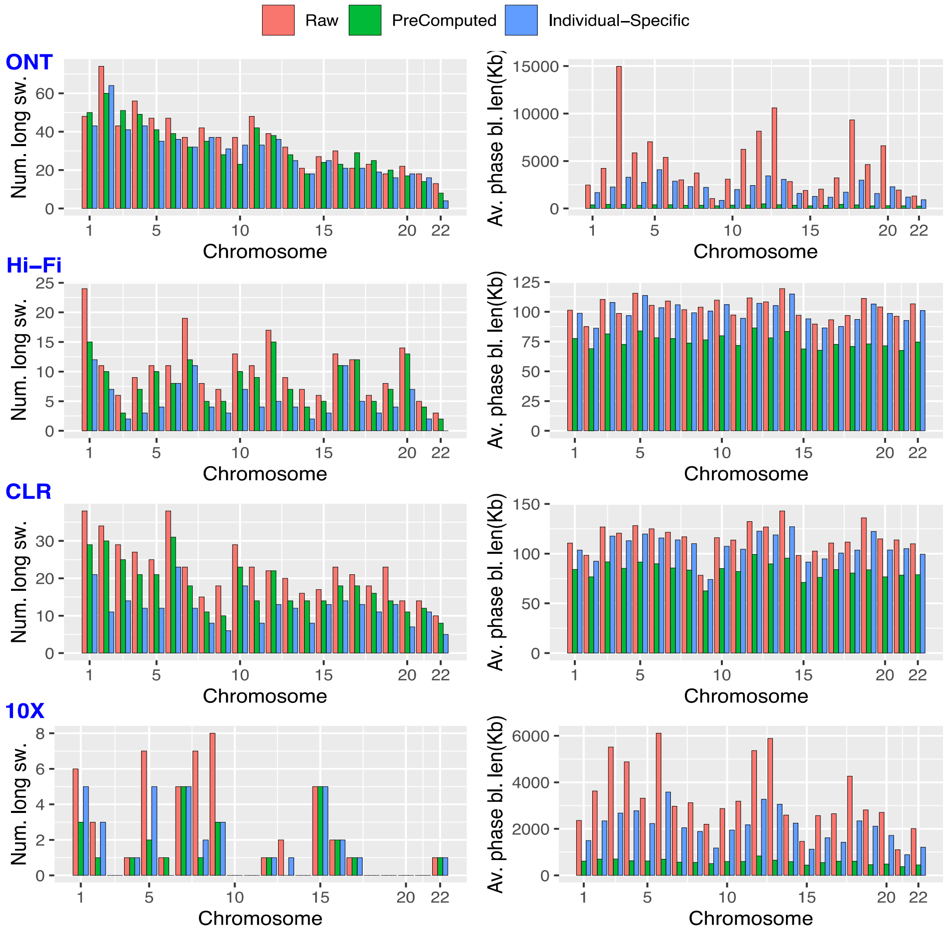

*Figure 3**: Phasing improvement.** Comparison of raw haplotypes and improved haplotypes in terms of number of long switches and average phase block length (kbp) for four datasets ONT, PacBio CLR, PacBio HiFi, and 10Xgenomics. Precomputed with EUR_AF≥0.01.*

	**Short switch**		**Long switch**		**Run time (min)**	
**Technology**	**Raw**	**change (%)**	**Raw**	**change(%)**	**QC step**	**Error correction step**
**ONT**	698	+6.6	780	-17.9	120	6
**PacBio CLR**	1,452	-22.4	497	-46.0	10	4
**PacBio HiFi**	455	-18.6	230	-54.6	9	4
**10Xgenomics**	1,356	-1.5	50	-23.5	135	7

***Table 1:** Summary of results using PhaseME including Short (2-20bp), Long (21+bp) switches and the corresponding run time in minutes A negative value indicates the improvement while a positive value represents an introduction of false cuts. For short switches, we observed a lower performance due to the low resolution of linkage data compared to the long switch errors. For the runtime the QC part takes longer as it includes the detection of the errors.*

To further ease the usage of PhaseME for non-expert users we implemented two modes. In general, we recommend following the instructions to obtain linkage information for each sample. Nevertheless, to ease the usage of PhaseME, we also investigated the usage of precomputed linkage maps. We computed the linkage map based on SNP for a given ethnicity (EUR_AF≥0.01). The number of long switch errors for PacBio HiFi and CLR are only improved by 22.6% and 20.9% while the average phase block length is 75.2kbp and 83.2kbp, respectively. For ONT PhaseME improved 10.6% of the number of long switch errors leading to an average phase block length of 355.1kbp. Further adjustments of the minimum allele frequency did not provide significant improvements (Supplementary Table 1 and 2). Therefore, we recommend using the precomputed linkage maps only for non-expert users but suggest following our guidelines to obtain a sample specific linkage map once larger errors are initially detected. PhaseME can compute a rapid quality assessment which run time dependents on phase block size. For these data sets, PhaseME took between nine minutes (HiFi) to 135

minutes (10Xgenomics) to compute the QC results, which includes error detection (see Table 1). For the error correction steps, PhaseME is optimized to run multiple times with different parameters and only requires between four minutes (HiFi) up to seven minutes (10Xgenomics).

PhaseME represents a versatile and easy to use method to obtain insights into the phasing performance independent of the underlying sequencing technology. It allows non-expert users to gain valuable insights into the data set and the correctness of the phasing given a linkage map is available. Here we have shown the performance of PhaseME based on HG002 across four different sequencing technologies demonstrating a significant improvement over long switch errors. Smaller errors in phasing remains a challenge due to the lower resolution of linkage data. Consequently, we did not attempt to correct single SNV phasing errors. To enable utility to a broader range of users we have provided precomputed linkage maps that can be used to obtain an initial improvement and insight, but highly recommend users to compute the linkage map specific to their study. PhaseME is capable of being run on multiple organisms and functions irrespective of the phasing method.

Methods

PhaseME Prerequisites

PhaseME requires phased VCF files as input, which needs to follow the VCF standards 4.1 or newer [31]. For processing, PhaseME requires the tags (PS and GT) to identify the phase blocks and genotypes such as 0|1 or 1|0 to indicate the haplotypes per SNV. PhaseME is written in Python3 and requires the Numpy package.

To exploit population information and obtain the linkage information, we used Shapeit2 (version v2r900) [27] based on the 1000 Genome dataset [23]. Since the Shapeit2 package needs phased data per chromosome, we split the input VCF file into 22 VCF files

corresponding to 22 chromosomes. We also removed non-genotyped variants from each VCF. Using the '-check' subprogram of Shapeit2 with '--input-vcf' option, we report the missing variants in the reference panel which is then excluded using '--exclude-snp' option of Shapeit2. Then we generate a haplotype graph which is a compact format of population information using the '--output-graph' option accompanying the genetic map with '-M' and phased haplotype, legend, and sample names with '--input-ref' options. Then using the '-convert' subprogram of Shapeit2, we sample the haplotype graph and generate haplotype samples (default 500 times). Subsequently, haplotype samples are used to generate a pair list. Each element of the list contains the positions of two SNVs and the relation of their phased GT. If the phasing of two variants in 90% samples are identical (or opposite), we will report them in the pair list.

Definition of quality criteria

Most of the measurements reported by PhaseME are self explained such as the number of heterozygous SNV, phased SNV, N50 phasing, and non-phased heterozygous SNV to give insights into the phasing performance. Nevertheless, a few metrics exist that we describe here in detail:

For calculating the average phase block quality, we averaged the ratio of the number of matched SNVs over the mismatched SNVs along each chromosome based on the linkage information. Phase rate is calculated by dividing the summation of phase block length by the difference in the position of last and first SNVs of the chromosome.

Data description

The raw reads were obtained from GIAB FTP (ONT ftp://ftp-trace.ncbi.nlm.nih.gov/giab/ftp/data/AshkenazimTrio/HG002_NA24385_son/UCSC_Ultralong_OxfordNanopore_Promethion, PacBio Hi-Fi ftp://ftp-trace.ncbi.nlm.nih.gov/giab/ftp/data/AshkenazimTrio/HG002_NA24385_son/PacBio_CCS_15kb/ and PacBio CLR ftp://ftp-trace.ncbi.nlm.nih.gov/giab/ftp/data/

AshkenazimTrio/HG002_NA24385_son/PacBio_MtSinai_NIST/). The ONT and PacBio reads were aligned using NGMLR [28] to the human reference genome (hg19). Subsequently, we identified SNV using the Clair2 package [29]. We used WhatsHap [21] to phase the SNV based on sequencing reads. The phased data of 10Xgenomics were downloaded from GIAB FTP (ftp://ftp-trace.ncbi.nlm.nih.gov/giab/ftp/data/AshkenazimTrio/analysis/10XGenomics_ChromiumGenome_LongRanger2.2_Supernova2.0.1_04122018/GRCh37/NA24385_LongRanger_snpindel.vcf.gz).

We downloaded 1000 Genome data phase 3, including 2504 samples [30]. The population information was downloaded from (https://mathgen.stats.ox.ac.uk/impute/1000GP_Phase3.tgz) including phased haplotype in IMPUTE format (that consists of 0s (reference allele) and 1s (alternate allele)), the legend file in IMPUTE format including the genomic position of variants, the reference and alternate allele in base, the allele frequency in populations, and the genetic map file in IMPUTE format of physical positions in NCBI b37 coordinates. The membership of each sample is reported in '1000GP_Phase3.sample' file.

<u>Benchmarking of phasing data</u>

Here we used parental information to benchmark the results of PhaseME. We have downloaded the high confidence parental call set from GIAB FTP (ftp://ftp-trace.ncbi.nlm.nih.gov/giab/ftp/release/AshkenazimTrio/HG003_NA24149_father/latest/GRCh37/HG003_GRCh37_GIAB_highconf_CG-IllFB-IllGATKHC-Ion-10X_CHROM1-22_v.3.3.2_highconf.vcf.gz and ftp://ftp-trace.ncbi.nlm.nih.gov/giab/ftp/release/AshkenazimTrio/HG004_NA24143_mother/latest/GRCh37/HG004_GRCh37_GIAB_highconf_CG-IllFB-IllGATKHC-Ion-10X_CHROM1-22_v.3.3.2_highconf.vcf.gz). For each technology, we generate the phased SNV VCF (see above) for the HG002 son. To generate the parental set, we first combine all the three call sets of the son (being the one to be benchmarked), mother and father using bcftools merge [31]. Then by considering the heterozygous SNVs, we generate a phasing set

for the son using in-house python code. Given the overlap between a het SNV of the son and to only the father, we report the phasing as 0|1. If it was overlapping exclusively with the mother, 1|0 is reported. The output VCF file is used for evaluation of the individual phasing method to calculate the Hamming error rate, the number of short and long switches using an in-house python code provided on our GitHub page.

The Hamming error rate is defined as the number of individual's phasings that are different from the true phasing divided by the number of phased heterozygous variants. A switch is defined by comparing the phased VCF with true. For each phase block we first compute the number of agreeing and disagreeing phasing information. If the majority of SNV are disagreeing, we need to consider that the phase block is reported the other way around and thus invert the result. Subsequently the remaining mismatching phase genotypes represent errors in the phasing *i.e.* switch errors if there are multiple in a row. The reported results are averaged over phase blocks.

Availability of supporting source code and requirements

Project name: PhaseME

Project home page: https://github.com/smajidian/phaseme

Operating system: Linux.

Programming language: Python

Other requirements: Python 3.6 or higher

License: The MIT License.

Availability of supporting data

The data set supporting the results of this article is available in the GigaDB repository, [last reference].

List of abbreviations

VCF: Variant Call Format; ONT: Oxford Nanopore Technologies; SNV: Single Nucleotide Variation; Thiopurine S-methyltransferase: TPMT; SIH: Single Individual Haplotyping; GIAB: Genome in a Bottle; bp: Base Pair; SV: Structural Variation; CLR: Continuous Long Read.

Ethics approval and consent to participate

Not applicable.

Consent for publication

Not applicable.

Competing interests

F.J.S. has participated in PacBio and Oxford Nanopore sponsored meetings over the past few years and has received travel reimbursement for presenting at these events. F.J.S. also received the SMRT PacBio sequencing grant in 2018.

Funding

This work was supported in part by the US National Institutes of Health (UM1 HG008898)

Authors' contributions

The project was conceived by F.J.S. Methods were designed by F.J.S and S.M. Algorithm code was implemented by S.M. All authors wrote the manuscript. F.J.S. supervised the project.

Acknowledgments

We would like to thank Shalini Jhangiani and Medhat Mahmoud for helpful discussions.

References

1. Sedlazeck FJ, Lee H, Darby CA, Schatz MC. Piercing the dark matter: bioinformatics of

long-range sequencing and mapping. Nat Rev Genet. 2018;19:329–46.

2. Choi Y, Chan AP, Kirkness E, Telenti A, Schork NJ. Comparison of phasing strategies for whole human genomes. PLoS Genet. 2018;14:e1007308.

3. Beck CR, Carvalho CMB, Akdemir ZC, Sedlazeck FJ, Song X, Meng Q, et al. Megabase Length Hypermutation Accompanies Human Structural Variation at 17p11.2. Cell. 2019;176:1310–24.e10.

4. Yang H, Spitz MR, Stewart DJ, Lu C, Gorlov IP, Wu X. ATM sequence variants associate with susceptibility to non-small cell lung cancer. Int J Cancer. 2007;121:2254–9.

5. Barroso E, Milne RL, Fernández LP, Zamora P, Arias JI, Benítez J, et al. FANCD2 associated with sporadic breast cancer risk. Carcinogenesis. 2006;27:1930–7.

6. Pelletier C, Speed WC, Paranjape T, Keane K, Blitzblau R, Hollestelle A, et al. RareBRCA1haplotypes including 3'UTR SNPs associated with breast cancer risk [Internet]. Cell Cycle. 2011. p. 90–9. Available from: http://dx.doi.org/10.4161/cc.10.1.14359

7. Leija-Salazar M, Sedlazeck FJ, Toffoli M, Mullin S, Mokretar K, Athanasopoulou M, et al. Evaluation of the detection of GBA missense mutations and other variants using the Oxford Nanopore MinION. Mol Genet Genomic Med. 2019;7:e564.

8. Tewhey R, Bansal V, Torkamani A, Topol EJ, Schork NJ. The importance of phase information for human genomics. Nat Rev Genet. 2011;12:215–23.

9. Liu P-Y, Zhang Y-Y, Lu Y, Long J-R, Shen H, Zhao L-J, et al. A survey of haplotype variants at several disease candidate genes: the importance of rare variants for complex diseases. J Med Genet. 2005;42:221–7.

10. Appell ML, Berg J, Duley J, Evans WE, Kennedy MA, Lennard L, et al. Nomenclature for alleles of the thiopurine methyltransferase gene. Pharmacogenet Genomics. 2013;23:242–8.

11. Almoguera B, Vazquez L, Connolly JJ, Bradfield J, Sleiman P, Keating B, et al. Imputation of TPMT defective alleles for the identification of patients with high-risk phenotypes. Front Genet. 2014;5:96.

12. Kurzawski M, Dziewanowski K, Ciechanowski K, Droździk M. Severe azathioprine-induced myelotoxicity in a kidney transplant patient with thiopurine S-methyltransferase-deficient genotype (TPMT*3A/*3C). Transpl Int. 2005;18:623–5.

13. Relling MV, Schwab M, Whirl-Carrillo M, Suarez-Kurtz G, Pui C-H, Stein CM, et al. Clinical Pharmacogenetics Implementation Consortium Guideline for Thiopurine Dosing Based on TPMT and NUDT15 Genotypes: 2018 Update. Clin Pharmacol Ther. 2019;105:1095–105.

14. Browning SR, Browning BL. Haplotype phasing: existing methods and new developments. Nat Rev Genet. 2011;12:703–14.

15. Huang M, Tu J, Lu Z. Recent Advances in Experimental Whole Genome Haplotyping Methods. Int J Mol Sci [Internet]. 2017;18. Available from: http://dx.doi.org/10.3390/ijms18091944

16. Ma L, Xiao Y, Huang H, Wang Q, Rao W, Feng Y, et al. Direct determination of molecular haplotypes by chromosome microdissection. Nat Methods. 2010;7:299–301.

17. Browning SR, Browning BL. Rapid and accurate haplotype phasing and missing-data inference for whole-genome association studies by use of localized haplotype clustering. Am J Hum Genet. 2007;81:1084–97.

18. Browning BL, Browning SR. A fast, powerful method for detecting identity by descent. Am J Hum Genet. 2011;88:173–82.

19. Garg S, Martin M, Marschall T. Read-based phasing of related individuals. Bioinformatics. 2016;32:i234–42.

20. Edge P, Bafna V, Bansal V. HapCUT2: robust and accurate haplotype assembly for

diverse sequencing technologies. Genome Res. 2017;27:801–12.

21. Martin M, Patterson M, Garg S, Fischer SO, Pisanti N, Klau GW, et al. WhatsHap: fast and accurate read-based phasing [Internet]. Available from: http://dx.doi.org/10.1101/085050

22. Bansal V. Integrating read-based and population-based phasing for dense and accurate haplotyping of individual genomes. Bioinformatics. 2019;35:i242–8.

23. Zook JM, Catoe D, McDaniel J, Vang L, Spies N, Sidow A, et al. Extensive sequencing of seven human genomes to characterize benchmark reference materials. Sci Data. 2016. p. 160025.

24. Zook JM, Hansen NF, Olson ND, Chapman LM, Mullikin JC, Xiao C, et al. A robust benchmark for germline structural variant detection [Internet]. bioRxiv. 2019 [cited 2020 Jan 21]. p. 664623. Available from: https://www.biorxiv.org/content/10.1101/664623v3.abstract

25. Wenger AM, Peluso P, Rowell WJ, Chang P-C, Hall RJ, Concepcion GT, et al. Accurate circular consensus long-read sequencing improves variant detection and assembly of a human genome. Nat Biotechnol. 2019;37:1155–62.

26. Porubsky D, Garg S, Sanders AD, Korbel JO, Guryev V, Lansdorp PM, et al. Dense and accurate whole-chromosome haplotyping of individual genomes. Nat Commun. 2017;8:1293.

27. Delaneau O, Zagury J-F, Marchini J. Improved whole-chromosome phasing for disease and population genetic studies. Nat Methods. 2013;10:5–6.

28. Sedlazeck FJ, Rescheneder P, Smolka M, Fang H, Nattestad M, von Haeseler A, et al. Accurate detection of complex structural variations using single-molecule sequencing. Nat Methods. 2018;15:461–8.

29. Luo R, Wong C-L, Wong Y-S, Tang C-I, Liu C-M, Leung C-M, et al. Clair: Exploring the limit of using a deep neural network on pileup data for germline variant calling [Internet]. Available from: http://dx.doi.org/10.1101/865782

30. 1000 Genomes Project Consortium, Auton A, Brooks LD, Durbin RM, Garrison EP, Kang HM, et al. A global reference for human genetic variation. Nature. 2015;526:68–74.

31. Li H. A statistical framework for SNP calling, mutation discovery, association mapping and population genetical parameter estimation from sequencing data. Bioinformatics. 2011;27:2987–93.

Additional files

Supplementary Table 1. Number of long switches for different thresholds using precomputed pair lists.

Supplementary Table 2. Number of short switches for different thresholds using precomputed pair lists.

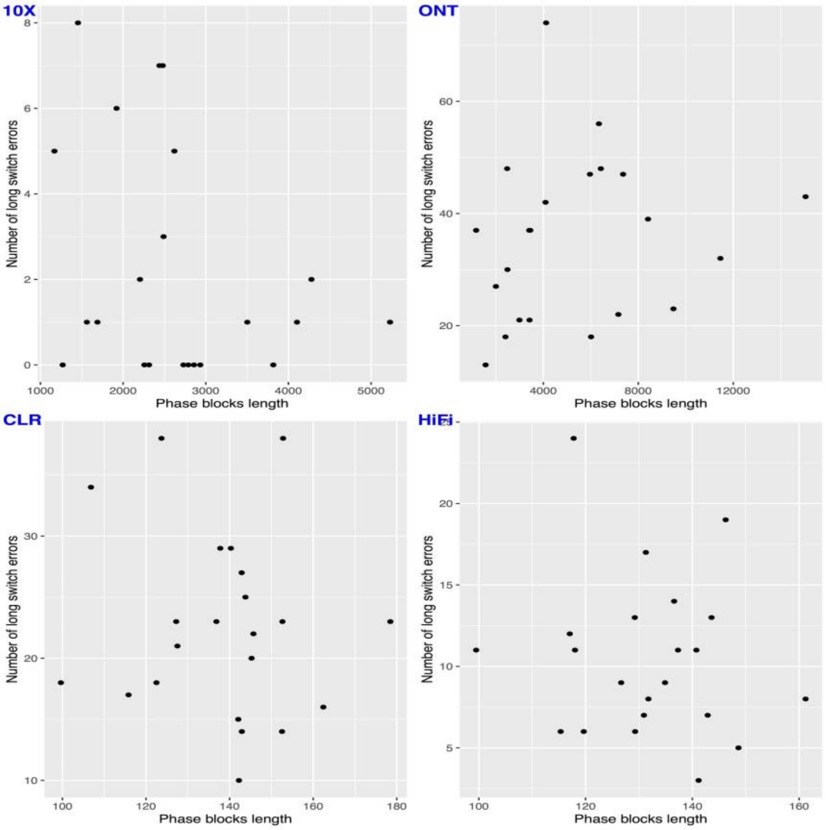

__Supplementary Figure 1.__ Number of long switch errors VS the length of phase blocks in which each point is a chromosome of four technologies. No correlation between the number of long switch errors and the length of phase blocks has been observed.

Supplementary Table

Click here to access/download
Supplementary Material
PhaseME_SuppTable.xlsx

Personal Cover Click here to download Personal Cover Cover_Letter.docx

April 3rd, 2020

Baylor College of Medicine
Human Genome Sequencing Center

One Baylor Plaza
Houston, Texas 77030-3498

TEL: (713)798-6539
FAX: (713)798-5741

Dear Editor,

Please find enclosed a copy of the manuscript “PhaseME: automatic rapid assessment of phasing quality and phasing improvement”, which we submit for consideration to *Gigascience*.

In our manuscript, we present the first method to assess phasing quality and improvements for single sample phasing. PhaseME provides an intuitive report to the expert and non-expert users for their phasing results. In an optional step, it can also improve the single sample phasing results.

As you know, phasing small variations (SNV) is essential to better understand the coexistence of two or more SNV on the same DNA molecule and therefore their potential impact on genes. While multiple methods have been developed to obtain this information based on *e.g.* long or linked reads, it remains a challenge to assess the quality of the results. The current assessment comparison only revolves around N50 and other length comparison or the need of parental data. This is impractical and remains a challenge. Thus we have developed PhaseME to solve this problem. We demonstrate the utility of PhaseME based on HG002 GAIB (NIST) data across variant calling and phasing results from PacBio (CLR & HiFi), ONT and 10xgenomics. PhaseME was able to detect multiple issues for each of the four sequencing technologies. Furthermore, PhaseME was able to improve the phasing results significantly, which was evaluated based on the reported gold standard from GIAB and parental data.

To ensure broad adoption, we provide detailed documentation on how to utilize PhaseME and provide two options for expert and non-expert users with precomputed linkage maps. Thus, we are confident that this work will have a high degree of impact, citations and is also of interest to the readers of Gigascience.

We further suggest potential reviewers based on their experience working on genomic data and involvement in developing methods around phasing: Tobias Marshall (tobias. marschall@hhu.de Max Planck Institute), Olivier Delaneau (olivier.delaneau@unige.ch University of Lausanne), Sorin Istrail (Sorin_Istrail@Brown.edu Brown University), Ryan Layer ryan.layer@colorado.edu University of Colorado), Gunnar W. Klau (gunnar.klau@hhu. de University Düsseldorf), Vineet Bafna (vbafna@ucsd.edu Hapcut, University of California, San Diego) and Vikas Bansal (vibansal@ucsd.edu, University of California, San Diego).

Warm Regards,

Fritz J. Sedlazeck, Ph.D.

Assistant Professor,
Human Genome Sequencing Center,
Baylor College of Medicine,
Houston, TX, 77030
