# Supplementary material for: PhaseME: Automatic rapid assessment of phasing quality and phasing improvement
Source: Gigascience. 2020 Jul 24;9(7):giaa078. doi: 10.1093/gigascience/giaa078 (PMC7379178; doi:10.1093/gigascience/giaa078)
Supplement: giaa078_Response_to_Reviewer_Comments_Original_Submission [file giaa078_response_to_reviewer_comments_original_submission.pdf]

Author's Response To Reviewer Comments

Close

We have attached a detailed and formatted response in the Cover letter for the resubmission.

We thank the reviewers for their positive feedback and the useful comments on the manuscript. We addressed all the comments and incorporated the suggestions and replies below and in the main text in red to make it easier to see the changes.

Reviewer #1: The authors presented a method that can both assess phasing completeness and improving switch errors based on linkage information. This will be a useful tool to quickly assess and improve variants phased from long-read alignments. However, besides the usefulness, a few critical descriptions are missing that needs to be addressed.

We thank the reviewer for his/her suggestions and highlighting the importance of PhaseME.

Major comments

1. In page 7, the authors found "a lower phase quality for chromosome 9 when using ONT and 10Xgenomics data". Why was it lower in these two technologies?

We investigated the source of this error for ONT and 10Xgenomics on chromosome 9. When we compared the SNV calling to the GIAB gold standard, we found lower precisions on the ONT (0.67) and 10Xgenomics (0.71) call sets which highlights a larger number of falsely called SNV for ONT (64,496 SNV) and 10Xgenomics (61,392 SNV). Whereas CLR (56,799 SNV) and HiFi (29,995 SNV) had lower false positive SNVs. These additional SNVs from ONT and 10Xgenomics on Chr9 are likely the cause of larger phasing errors. We have added the mentioned description to page 8 of the text and provided the detailed results in Supplementary Table 3.

2. Phase correction results seem to be contradictory and inconclusive, with little explanations. Why is the phase block N50 decreasing after correction? After correcting long-range switches, the block N50s are expected to increase, not decrease.

PhaseME is designed to correct phasing errors (mainly larger switching errors) by cutting the phase blocks into two pieces. This halves the overall length of the phaseblock and thus the phasing N50 is impacted. PhaseME does not attempt to join different phaseblocks together as this can also introduce biases based on population data (e.g. at recombination sites).

3. Related to 2., how is the phase correction performed? Method is completely missing how the phase blocks are broken and rearranged during correction.

The current version is very conservative and the phase blocks are simply broken (described in the first paragraph of "Phasing error correction" Section.). We experimented around with fixing these orientations but the results did not greatly improve likely due to the resolution of the population data.

4. The authors argue single individual haplotyping with linkage information is more affordable than using parental information. This is true only when the linkage information is already available for the ancestral population of the sequenced individual. Obtaining linkage information is more expensive when it scales to hundreds of people compared to sequencing the parental genomes.

This is correct. We adopted the text also including reviewers 2 comment that by this we probably limit ourselves to model organisms as large population sequencing projects of non model organisms are often not existing. We further added a mode to utilize parental SNV information to assess the quality of phasing.

5. Page 10, second paragraph is very confusing. Why should the number of long switch errors and the length of phase blocks expected to correlate? What is their relationship to the phase block accuracy (which is measured with consistency to linkage information) and hamming error rate?

An extreme case would be when we compare 1 phase block spanning the entire chromosome arm vs. other phase blocks that span only a few SNVs (e.g. 3 SNVs). The risk/probability that an inaccuracy (e.g. in the SNV calling or phasing itself) was incorporated is higher in the larger phase block given that it spans also regions of low complexity or low heterozygosity etc. Clearly this is dependent on the sequencing technology and phasing method that is used.

6. Authors show in their results that linkage data does not provide sufficient resolution to correct single flip errors or short-range switch errors. For a broad interest to apply this method on clinical data, it will be better to consider implementing a way to evaluate and correct based on parental genotyping information. De novo mutations will not be able to evaluate, however, will have a much higher resolution compared to linkage data. I'd argue most clinical data needs higher resolution, and have often parental or familial genotyping information available, to correctly detect compound heterozygosity. It will be also useful to flag do novo mutation-like switches for further investigation.

We appreciate the suggestion and incorporated this mode also in PhaseME that it can also leverage parental information when available. We briefly mentioned this now also in the main text.

7. A distinction needs to be made between variants called by 'error', due to the noisy reads, which are un-classified (not phased). These regions should be included when measuring phasing completeness.

Yes, we have included SNVs and indels that were identified based on the long and linked reads without any filtering for GIAB regions or SNV. So phasing completeness represents all variations that were identified for each technology respectively.

8. What is the difference between hamming error rate per phase blocks and the phase block quality used in PhaseMe? I'd argue just to use the hamming error rate per blocks.

The reason why we did not use hamming error is that the linkage information has limited resolution on small errors (i.e. flip). We have now incorporated the mode to utilize parental information in which case the hamming error and phase block quality will be more similar.

Minor comments
1. In the abstract, page 1: "a high N50 and completeness" of what? is missing here.

We made that correction.

2. Page 2, emphasize the use of long-reads to cite reference [1].

We added multiple other references to highlight this also outside of the long read review.

3. Page 3, remove "more expensive" in 3. Parental methods. This is true only when the linkage information is already available for the ancestral population of the sequenced individual. Obtaining linkage information is more expensive when it scales to hundreds of people compared to sequencing the parental genomes, when considering consenting, extracting DNA, genotyping, and associated labor work for each individual.

We edited part 3. Moreover, we adopted this sentence to part 2 in page 3 : “Furthermore, it requires available population based sequencing which might not be available for many of the non-model organisms”.

4. Page 6, the accuracy value for 0 to 1 seems the other way around compared to what was described in the above line: "non-conflicting to conflicting linkage information". The value also should go over 1 when there are more non-conflicting sites. Please correct the sentence or give the equation to clarify how the values between 0 and 1 were computed.

You are right. We are using the total number of SNVs per phase block in the equation. This was corrected in the text.

5. Page 6, subheading Quality Control (QC) seems inappropriate as PhaseMe is not controlling the phasing results in anyway. "Quality assessment" might be a better term to describe the functions PhaseMe is performing.

We made that correction: “Quality assessment based on PhaseME”

6. Page 7, iii) is missing.
We made that correction.

7. Page 7, between the first and second paragraph: Add a brief description how the phase blocks were generated. At least, "What" was aligned to "which reference version", "how" the variants were called and phased needs to be stated in before the results.

These details were reported in the Methods Section: "The ONT and PacBio reads were aligned using NGMLR [28] to the human reference genome (hg19). Subsequently, we identified SNV using the Clair2 package [29]. We used WhatsHap [21] to phase the SNV based on sequencing reads.". We did extend the text now for the Illumina based phasing. Furthermore, the Data description section has all the details of the used datasets.

8. Page 10, first paragraph, remove overlapping description in "Quality Control" part and clarify what has been corrected and benchmarked in which way.

This has been corrected. The details were outlined in the method section: Benchmarking of phasing data. In brief, we utilized the SNV available from the GIAB consortia from the parents (HG003 + HG004) using bcftools merge together with the per sample phasing information. This was done to assess the correctness of overall phasing from PhaseME.

Reviewer #2: The authors presents a method (PhaseME) that allows to assess the quality of phasing obtained from SIH methods, by providing a report with summary statistics and an (optional) vcf with the corrected switch errors. It is an interesting method and the software is user-friendly, but I had a problem when using the qc option, since the QC file obtained was empty (it worked well for improver option). I strongly recommend to improve the writing and to reoganize some sections. Most of the doubts I had when reading the manuscript were solved in other sections.

I have some general comments and I also detail more specific suggestions to improve the writing.

We thank the reviewer for his/her suggestions and highlighting the user friendliness and importance of PhaseME. We are sorry for the inconvenience about the QC summary file, which was written into a subfolder. This bug has been fixed.

The benchmarking was done by comparing with another computational method (parenting), which may suffer from other biases. I suggest to compare PhaseME results with experimentally validated phasing information, which is available for some species (e.g. Candida albicans).

We agree that parental information sometimes is not optimal, but is regarded as the most comprehensive phasing method. We added a sentence in the introduction cautioning the reader that parental information could also cause problems.: “ It is worth noting that parental data also has some limitations (e.g. de novo mutations), however HG002 is a healthy individual that is well studied.”

I also wonder how many individuals are needed to obtain accurate linkage maps. It may seem out of the scope of this paper, but is an important question since depending on the number needed it may limit its usage for several species.

This is a great question. It indeed limits the usability for PhaseME for many current non model organisms. The minimum number of individuals needed would depend on the population characteristics (bottlenecks, mutations and recombination rates), so we cannot find a clear answer to this question. Given your and reviewer1 suggestion we now also implemented a parental mode to utilize this information as well if available, which makes it more applicable for non model organisms as well.

Here only long reads are tested. Despite phasing with short reads is very challenging, tons of data are

available only for short reads. I may be interesting to quantify the quality of short reads phasing methods.

We ran the experiment also on Illumina reads using the xatlas package for SNV calling and WhatsHap for phasing with 69x coverage Illumina paired end reads. We have added the results to the manuscript. We observed a high phase block quality for Illumina (0.9936) very near to that of HiFi (0.994). However, as we expected due the size of Illumina reads the N50 is very low (1.3kbp) compared to HiFi (314.3kbp) and other long read technologies.

Title "Quality control" is misleading. It is not a quality control of the method but a comparison of different sequencing technologies. I was expecting to see a benchmarking of the method before of this comparison.

The term "Quality control" was used here for describing what can be found when running PhaseMe on the results to assess the quality. We renamed this in the manuscript to "Quality assessment using PhaseME". We followed the flow of the method to first use PhaseMe to obtain insights in the phasing performance. Here no benchmarks are required as it is just descriptive. Subsequently, we describe the identification of errors and how PhaseMe improves the results where we included benchmarks as this relies on a few assumptions.

Page 6: "We evaluate the accuracy of SNV phasing across ONT, PacBio CLR, PacBio HiFi [25] and 10Xgenomics." How was obtained the SNP calling and phasing of this data? No software nor citation is indicated in the results section, and this important for the reader to know. If different software was used (not only for phasing but also SNPcalling), conclusions are not strong.

The details of this were listed in the methods section (Header: Data description). We also improved this description in the main text. In short we used parental data obtained for GIAB and merged the parental VCF files with the per sample phased information using bcftools merge. This merged 3 sample VCF file was used to evaluate the performance.

"However, our comparisons to the parents indicated that for ONT we actually introduced 6.6% of short switch errors." → indicate that Table 1 contains a table with the percentage of short/long errors introduced by phaseME.

We made that correction.

Please specify the resources used for the computation time.

We added this to the main text: “The program is run on a Linux machine with 16GiB RAM using a single CPU (AMD 1.4 GHz) but typically requires not more than 3GiB of memory and a single core.”

Figure 1 should be modified to make it more informative. In the footnote says that PhaseME consists on three steps, which should be clear in the figure (by grouping blocks). It should clearly indicate which are the input and output files, and for the later ones to specify which are optional.

We made that correction. We also changed Quality Control to Quality Assessment to avoid this confusion again.

Please rephrase last sentence of the abstract for clarity (at least some brackets are missing): "Additionally, a significant improvement is obtained in the number of long switch errors especially for HiFi 54.6% with only a 5% decrease in phase block N50"

We rephrased that sentence.

"PhaseME is a novel method to assess the quality of phasing results and provide a quality control report. It also reduces phasing errors by exploiting population information." → this sentence is repetitive with the previous paragraph.

We fixed this in the main text.

Close
