# Supplementary material for: PhaseME: Automatic rapid assessment of phasing quality and phasing improvement
Source: Gigascience. 2020 Jul 24;9(7):giaa078. doi: 10.1093/gigascience/giaa078 (PMC7379178; doi:10.1093/gigascience/giaa078)
Supplement: giaa078_Response_to_Reviewer_Comments_Revision_1 [file giaa078_response_to_reviewer_comments_revision_1.pdf]

Author's Response To Reviewer Comments

Close

We thank the editor and the reviewers again for the positive feedback. Below are the few remaining items that we addressed in this version.

R1.Please consider the (very minor) final comment of reviewer 1.
Reviewer #1: One minor comment for improving readability is to moving the "We did not observe a correlation between the number of long switch errors and the length of phase blocks ... " sentence on page 10 to the end of the paragraph.

A1. We incorporated this suggestion.

R2. Our data curators will contact you shortly to prepare the supporting GigaDB repository. If you have the citation details, please include this to the reference list (including also the DOI link to the bibliography).

A2. We have been working with them and obtained a link that is now included in the manuscript.

R3. Please register your software application in the bio.tools and SciCrunch.org databases to receive RRID (Research Resource Identification Initiative ID) and biotoolsID identifiers, and include these in your manuscript, in the "source code availability section". This will facilitate tracking, reproducibility and re-use of your tool.

A3. The software has been registered and the link incorporated into the manuscript.

R4. Please add ORCIDs for both authors to the title page, if available.

A4. They have been placed next to the author names.

R5. When you first mention the data from the GIAB FTP (in the data description section, page 16), please include a citation in the bibliography to the GIAB project site or the "top level" domain of the FTP sites that are mentioned in this section.

A5. This has been included.

R6. Also URLs (such as from software project sites, data sources etc.) should be items in the bibliography and cited by number in the text.

A6. we incorporated all links into the references.

R7. At this stage, please remove any highlighting/tracking in red that was made for the purpose of peer review.

A7. This has been done.

Close
