# Supplementary material for: PhaseME: Automatic rapid assessment of phasing quality and phasing improvement
Source: Gigascience. 2020 Jul 24;9(7):giaa078. doi: 10.1093/gigascience/giaa078 (PMC7379178; doi:10.1093/gigascience/giaa078)
Supplement: giaa078_Reviewer_1_Report_Original_Submission — Arang Rhie -- 5/5/2020 Reviewed [file giaa078_reviewer_1_report_original_submission.pdf]

Reviewer Report

Title: PhaseME: automatic rapid assessment of phasing quality and phasing improvement

Version: Original Submission **Date:** 5/5/2020

Reviewer name: Arang Rhie

Reviewer Comments to Author:

The authors presented a method that can both assess phasing completeness and improving switch errors based on linkage information. This will be a useful tool to quickly assess and improve variants phased from long-read alignments. However, besides the usefulness, a few critical descriptions are missing that needs to be addressed.

Major comments

1. In page 7, the authors found "a lower phase quality for chromosome 9 when using ONT and 10Xgenomics data". Why was it lower in these two technologies?
2. Phase correction results seem to be contradictory and inconclusive, with little explanations. Why is the phase block N50 decreasing after correction? After correcting long-range switches, the block N50s are expected to increase, not decrease.
3. Related to 2., how is the phase correction performed? Method is completely missing how the phase blocks are broken and rearranged during correction.
4. The authors argue single individual haplotyping with linkage information is more affordable than using parental information. This is true only when the linkage information is already available for the ancestral population of the sequenced individual. Obtaining linkage information is more expensive when it scales to hundreds of people compared to sequencing the parental genomes.
5. Page 10, second paragraph is very confusing. Why should the number of long switch errors and the length of phase blocks expected to correlate? What is their relationship to the phase block accuracy (which is measured with consistency to linkage information) and hamming error rate?
6. Authors show in their results that linkage data does not provide sufficient resolution to correct single flip errors or short-range switch errors. For a broad interest to apply this method on clinical data, it will be better to consider implementing a way to evaluate and correct based on parental genotyping information. De novo mutations will not be able to evaluate, however, will have a much higher resolution compared to linkage data. I'd argue most clinical data needs higher resolution, and have often parental or familial genotyping information available, to correctly detect compound heterozygosity. It will be also useful to flag do novo mutation-like switches for further investigation.
7. A distinction needs to be made between variants called by 'error', due to the noisy reads, which are un-classified (not phased). These regions should be included when measuring phasing completeness.
8. What is the difference between hamming error rate per phase blocks and the phase block quality used in PhaseMe? I'd argue just to use the hamming error rate per blocks.

Minor comments

1. In the abstract, page 1: "a high N50 and completeness" of what? is missing here.
2. Page 2, emphasize the use of long-reads to cite reference [1].

3. Page 3, remove "more expensive" in 3. Parental methods. This is true only when the linkage information is already available for the ancestral population of the sequenced individual. Obtaining linkage information is more expensive when it scales to hundreds of people compared to sequencing the parental genomes, when considering consenting, extracting DNA, genotyping, and associated labor work for each individual.
4. Page 6, the accuracy value for 0 to 1 seems the other way around compared to what was described in the above line: "non-conflicting to conflicting linkage information". The value also should go over 1 when there are more non-conflicting sites. Please correct the sentence or give the equation to clarify how the values between 0 and 1 were computed.
5. Page 6, subheading Quality Control (QC) seems inappropriate as PhaseMe is not controlling the phasing results in anyway. "Quality assessment" might be a better term to describe the functions PhaseMe is performing.
6. Page 7, iii) is missing.
7. Page 7, between the first and second paragraph: Add a brief description how the phase blocks were generated. At least, "What" was aligned to "which reference version", "how" the variants were called and phased needs to be stated in before the results.
8. Page 10, first paragraph, remove overlapping description in "Quality Control" part and clarify what has been corrected and benchmarked in which way.

Level of Interest

Please indicate how interesting you found the manuscript: Choose an item.

Quality of Written English

Please indicate the quality of language in the manuscript: Choose an item.

Declaration of Competing Interests

Please complete a declaration of competing interests, considering the following questions:

- Have you in the past five years received reimbursements, fees, funding, or salary from an organisation that may in any way gain or lose financially from the publication of this manuscript, either now or in the future?
- Do you hold any stocks or shares in an organisation that may in any way gain or lose financially from the publication of this manuscript, either now or in the future?
- Do you hold or are you currently applying for any patents relating to the content of the manuscript?
- Have you received reimbursements, fees, funding, or salary from an organization that holds or has applied for patents relating to the content of the manuscript?
- Do you have any other financial competing interests?

- Do you have any non-financial competing interests in relation to this paper?

If you can answer no to all of the above, write 'I declare that I have no competing interests' below. If your reply is yes to any, please give details below.

I declare that I have no competing interests.

I agree to the open peer review policy of the journal. I understand that my name will be included on my report to the authors and, if the manuscript is accepted for publication, my named report including any attachments I upload will be posted on the website along with the authors' responses. I agree for my report to be made available under an Open Access Creative Commons CC-BY license (http://creativecommons.org/licenses/by/4.0/). I understand that any comments which I do not wish to be included in my named report can be included as confidential comments to the editors, which will not be published.

Choose an item.

To further support our reviewers, we have joined with Publons, where you can gain additional credit to further highlight your hard work (see: https://publons.com/journal/530/gigascience). On publication of this paper, your review will be automatically added to Publons, you can then choose whether or not to claim your Publons credit. I understand this statement.

Yes Choose an item.
