# Supplementary material for: PhaseME: Automatic rapid assessment of phasing quality and phasing improvement
Source: Gigascience. 2020 Jul 24;9(7):giaa078. doi: 10.1093/gigascience/giaa078 (PMC7379178; doi:10.1093/gigascience/giaa078)
Supplement: giaa078_Reviewer_2_Report_Original_Submission — Cinta Pegueroles -- 5/9/2020 Reviewed [file giaa078_reviewer_2_report_original_submission.pdf]

**Reviewer Report**

**Title: PhaseME: automatic rapid assessment of phasing quality and phasing improvement**

**Version: Original Submission** **Date:** 5/9/2020

**Reviewer name: Cinta Pegueroles**

**Reviewer Comments to Author:**

The authors presents a method (PhaseME) that allows to assess the quality of phasing obtained from SIH methods, by providing a report with summary statistics and an (optional) vcf with the corrected switch errors. It is an interesting method and the software is user-friendly, but I had a problem when using the qc option, since the QC file obtained was empty (it worked well for improver option). I strongly recommend to improve the writing and to reoganize some sections. Most of the doubts I had when reading the manuscript were solved in other sections.
I have some general comments and I also detail more specific suggestions to improve the writing.
The benchmarking was done by comparing with another computational method (parenting), which may suffer from other biases. I suggest to compare PhaseME results with experimentally validated phasing information, which is available for some species (e.g. Candida albicans).
I also wonder how many individuals are needed to obtain accurate linkage maps. It may seem out of the scope of this paper, but is is an important question since depending on the number needed it may limit its usage for several species.
Here only long reads are tested. Despite phasing with short reads is very challenging, tons of data are available only for short reads. I may be interesting to quantify the quality of short reads phasing methods.
Title "Quality control" is misleading. It is not a quality control of the method but a comparison of different sequencing technologies. I was expecting to see a benchmarking of the method before of this comparison.
Page 6: "We evaluate the accuracy of SNV phasing across ONT, PacBio CLR, PacBio HiFi [25] and 10Xgenomics." How was obtained the SNP calling and phasing of this data? No software nor citation is indicated in the results section, and this important for the reader to know. If different software was used (not only for phasing but also SNPcalling), conclusions are not strong.
"However, our comparisons to the parents indicated that for ONT we actually introduced 6.6% of short switch errors." â†' indicate that Table 1 contains a table with the percentage of short/long errors introduced by phaseME.
Please specify the resources used for the computation time.
Figure 1 should be modified to make it more informative. In the footnote says that PhaseME consists on three steps, which should be clear in the figure (by grouping blocks). It should clearly indicate which are the input and output files, and for the later ones to specify which are optional.
Please rephrase last sentence of the abstract for clarity (at least some brackets are missing):
"Additionally, a significant improvement is obtained in the number of long switch errors especially for HiFi 54.6% with only a 5% decrease in phase block N50"

"PhaseME is a novel method to assess the quality of phasing results and provide a quality control report. It also reduces phasing errors by exploiting population information." â†’ this sentence is repetitive with the previous paragraph.

**Level of Interest**

Please indicate how interesting you found the manuscript: Choose an item.

**Quality of Written English**

Please indicate the quality of language in the manuscript: Choose an item.

**Declaration of Competing Interests**

Please complete a declaration of competing interests, considering the following questions:

- Have you in the past five years received reimbursements, fees, funding, or salary from an organisation that may in any way gain or lose financially from the publication of this manuscript, either now or in the future?
- Do you hold any stocks or shares in an organisation that may in any way gain or lose financially from the publication of this manuscript, either now or in the future?
- Do you hold or are you currently applying for any patents relating to the content of the manuscript?
- Have you received reimbursements, fees, funding, or salary from an organization that holds or has applied for patents relating to the content of the manuscript?
- Do you have any other financial competing interests?
- Do you have any non-financial competing interests in relation to this paper?

If you can answer no to all of the above, write 'I declare that I have no competing interests' below. If your reply is yes to any, please give details below.

I declare that I have no competing interests

I agree to the open peer review policy of the journal. I understand that my name will be included on my report to the authors and, if the manuscript is accepted for publication, my named report including any attachments I upload will be posted on the website along with the authors' responses. I agree for my report to be made available under an Open Access Creative Commons CC-BY license (http://creativecommons.org/licenses/by/4.0/). I understand that any comments which I do not wish to be included in my named report can be included as confidential comments to the editors, which will not be published.

Choose an item.

To further support our reviewers, we have joined with Publons, where you can gain additional credit to further highlight your hard work (see: https://publons.com/journal/530/gigascience). On publication of this paper, your review will be automatically added to Publons, you can then choose whether or not to claim your Publons credit. I understand this statement.

Yes Choose an item.
